# Neuroimmune activation of the TRPV1–RAMP1 axis drives type 2 immunity to repair intestinal barrier and alleviate metabolic disease

**DOI:** 10.3389/fimmu.2026.1965520

**Published:** 2026-10-07

**Authors:** Xingyu Yang, Xiao Li, Yue Wu, Li An, Linglong Zhang, Ziwei Yu, Xinyue Jing, Tiancheng Xu, Zhi Yu, Jinhong Yuan, Bin Xu, Mengjiang Lu

**Affiliations:** 1 Key Laboratory of Acupuncture and Medicine Research of Ministry of Education, Nanjing University of Chinese Medicine, Nanjing, Jiangsu, China; 2 School of Traditional Chinese Medicine, Nanjing University of Chinese Medicine, Nanjing, China

**Keywords:** electroacupuncture, ILC2, intestinal inflammation, obesity, TRPV1

## Abstract

**Introduction:**

Obesity is closely associated with intestinal barrier dysfunction and chronic inflammation. Group 2 innate lymphoid cells (ILC2s) can receive and integrate signals from neuropeptides, neurotransmitters, and epithelial cells, thereby regulating local tissue inflammatory responses and promoting repair. However, the specific mechanism by which the nociceptor Transient Receptor Potential Vanilloid 1 (TRPV1) interacts with ILC2s to regulate intestinal homeostasis in obesity remains unclear.

**Objective:**

This study aimed to investigate whether electroacupuncture (EA) ameliorated obesity-mediated intestinal injury by modulating type 2 immune responses via the TRPV1- calcitonin gene-related peptide (CGRP)- receptor activity-modifying protein 1 (RAMP1) axis.

**Methods:**

A high-fat diet-induced obese mouse model was employed. The expression levels of TRPV1 and CGRP, the abundance of ILC2s, and the levels of type 2 cytokines IL-5 and IL-13 in colon tissues were assessed using flow cytometry, immunofluorescence staining, and Western blotting. Intestinal pathological changes and goblet cell status were evaluated via hematoxylin and eosin staining and Alcian blue-periodic acid-Schiff staining. Mechanistic verification was performed by specific ablation of TRPV1^+^ dorsal root ganglion neurons and pharmacological inhibition of the CGRP receptor or RAMP1.

**Results:**

EA treatment significantly increased CGRP derived from TRPV1-expressing sensory neurons, which subsequently activated type 2 immunity, as evidenced by the expansion of ILC2s and upregulation of related cytokines. This immune activation ultimately promoted the differentiation and proliferation of goblet cells and ameliorated high-fat diet-induced intestinal barrier damage. Notably, in mice with specific ablation of TRPV1^+^ dorsal root ganglion neurons, or treatment with a CGRP receptor antagonist, or ablation of intestinal ILC2s, EA failed to induce intestinal type 2 immune activation or alleviate diet-induced intestinal injury.

**Conclusion:**

Our findings demonstrate that electroacupuncture activates intestinal type 2 immunity via the TRPV1-CGRP-RAMP1 axis, identifying this pathway as a crucial mechanism for repairing obesity-associated intestinal barrier dysfunction.

## Introduction

Obesity is a chronic metabolic disease characterized by excessive adipose tissue deposition due to an imbalance between energy intake and expenditure. It is often accompanied by metabolic disorders and systemic chronic low-grade inflammation. Obesity has become a major global public health challenge, contributing to approximately 3.7 million annual deaths from related complications, including cardiovascular diseases, diabetes, and digestive system diseases (1) High-fat diet-induced obesity increases intestinal barrier permeability, resulting in the translocation of pathogenic microorganisms and other harmful substances. This process activates pattern recognition receptors on intestinal epithelial cells and immune cells, triggering innate immunity and the release of pro-inflammatory mediators. These pro-inflammatory cytokines further damage the intestinal barrier and increase intestinal permeability, promoting increased leakage of luminal microbes and dietary antigens. This cascade elicits both innate and adaptive intestinal immune responses, ultimately leading to persistent chronic low-grade intestinal inflammation and systemic metabolic dysregulation. Inflammatory cytokines can also alter intestinal epithelial cell turnover and gut hormone secretion, thereby affecting systemic blood glucose levels (2). Intestinal inflammation induced by a high-fat diet further affects multiple metabolic organs, ultimately contributing to the development of obesity-related metabolic disorders.

The intestine receives rich innervation, which can be classified into intrinsic neurons and extrinsic neurons. The cell bodies of intrinsic neurons are located in the myenteric plexus and submucosal plexus of the gut, forming the enteric nervous system (ENS), which mediates intestinal sensation, peristalsis, and the secretion of enzymes necessary for digestion independently of the central nervous system. Extrinsic neurons connect the gut to the spinal cord and brain and are further subdivided into extrinsic afferent sensory neurons, which transmit sensory information from the gut to the central nervous system, and extrinsic autonomic neurons, which innervate and regulate the gut and the ENS. Extrinsic afferent neurons can be further divided into vagal afferent neurons originating from the nodose ganglion (NG) and jugular ganglion (VG), and spinal afferent neurons originating from the dorsal root ganglia (DRG). Vagal afferent neurons project centrally to the brainstem, and their peripheral axons project via the vagus nerve to the small intestine and proximal colon. Spinal afferent neurons innervate the entire intestinal tract: thoracolumbar spinal afferent neurons innervate the small intestine and proximal colon, while lumbosacral spinal afferent neurons innervate the distal colon and rectum. Extrinsic autonomic neurons comprise sympathetic and parasympathetic nerves. Sympathetic efferent nerves from the thoracolumbar spinal cord project to the paravertebral and prevertebral ganglia, where they synapse with noradrenergic postganglionic neurons that innervate the gut (3–6). Among these, sympathetic nerves can act on β2-adrenergic receptors on group 2 innate lymphoid cells (ILC2s) in the small intestine, reducing ILC2 proliferation and the secretion of IL-5 and IL-13 (7). Intestinal transient receptor potential vanilloid 1 (TRPV1) is predominantly expressed in extrinsic primary afferent neurons innervating the colon, with cell bodies located in the DRG and peripheral terminals distributed in the colonic myenteric plexus and crypts. It can sense tissue damage, thereby driving avoidance behavior and the local release of neuropeptides from peripheral nerve endings (8). During DSS-induced colitis, chemogenetic activation of TRPV1^+^ neurons alleviates weight loss, colon shortening, mucosal structural damage, and ulcer formation. Conversely, silencing or genetic ablation of TRPV1^+^ neurons significantly exacerbates these pathological phenotypes, including increased infiltration of immune cells (macrophages and neutrophils). Supplementation with TRPV1-related neuropeptides, such as substance P and calcitonin gene-related peptide (CGRP), rescues this phenotype (9, 10), demonstrating that the protective effect of TRPV1 depends on downstream signaling. However, whether TRPV1 contributes to intestinal repair during obesity remains unclear.

Group 2 innate lymphoid cells (ILC2s) are core effector cells of intestinal type 2 immunity and are abundantly distributed in barrier tissues. They integrate signals from neuropeptides, neurotransmitters, and the epithelium to regulate local tissue inflammation and maintain tissue homeostasis. Their activity in the gut is regulated by tissue-specific signals. For instance, neuromedin U (NMU) produced by cholinergic neurons in the enteric nervous system triggers ILC2 activation (11), whereas signaling through the β2-adrenergic receptor suppresses ILC2 activity in the intestine during helminth infection (7). Under homeostatic conditions, TRPV1-expressing neurons originating from the dorsal root ganglia (DRG) maintain ILC2s in a low-activation state via local release of calcitonin gene-related peptide (α-CGRP) (12). However, whether TRPV1-ILC2 interactions contribute to repairing intestinal damage and maintaining homeostasis in the context of obesity remains unclear.

EA therapy demonstrates good clinical efficacy in treating obesity and gastrointestinal diseases (13), and it possesses dual effects of weight reduction and intestinal barrier protection with a favorable safety profile. The mechanisms underlying acupuncture effects are often related to neuro-immune regulation. TRPV1, a polymodal receptor expressed on sensory nerve endings in the skin, subcutaneous tissues, and mucosa, is an important target molecule for acupuncture stimulation (14). Regulating TRPV1 activity or its mediated neuropeptide signaling ultimately acts on various immune cells, providing new strategies for treating immune-related diseases such as chronic inflammation and allergic diseases.

In this study, we demonstrate that under obese conditions, EA activates a type 2 immune response via CGRP derived from TRPV1-expressing sensory nerve fibers in the intestine binding to receptor activity-modifying protein 1(RAMP1) on ILC2s, promoting goblet cell expression and intestinal repair. Deficiency of TRPV1 or RAMP1 led to suppression of the intestinal type 2 immune response and exacerbated intestinal injury in obese mice. Further research identified TRPV1^+^ DRG nociceptors as the key neuronal subpopulation mediating the electroacupuncture-induced intestinal type 2 immune response. These findings indicate that in obesity, electroacupuncture protects the intestinal mucosal barrier by activating the intestinal type 2 immune response via TRPV1-expressing neurons.

## Methods

### Animals

Five-week-old specific pathogen-free (SPF) male C57BL/6 mice were used in this study. All mice (a total of 298 mice) were housed under a 12-hour light/dark cycle in a temperature-and humidity-controlled SPF environment. All experimental procedures were strictly performed in accordance with the Principles of Laboratory Animal Care and the Guide for the Care and Use of Laboratory Animals issued by the Chinese National Science Council (Animal Ethics Approval Number: 202412A015).

### Anesthesia and euthanasia

Mice were anesthetized with 2% isoflurane via a nose-cone induction chamber. Anesthesia was maintained by continuous inhalation of the same gas mixture throughout the procedure, the depth of anesthesia was periodically verified by the absence of a pedal withdrawal reflex. At the end of the experiments, mice were euthanized by cervical dislocation. All personnel performing this procedure were adequately trained and proficient. The method was restricted to mice weighing less than 200 g and was approved by the Institutional Animal Care and Use Committee (IACUC).

### Animal husbandry

After one week of acclimatization, C57BL/6 mice were randomly divided into a normal diet (ND) group and a high-fat diet (HFD) group using a random number table. The ND group was fed a maintenance diet (containing 11.1% fat, 21.5% protein, and 67.4% carbohydrate) provided by the Animal Center of Nanjing University of Chinese Medicine. The HFD group was fed a high-fat diet (purchased from Suzhou Shuangshi Company) with an energy composition of 60% fat, 19% protein, and 21% carbohydrate. After 10 weeks of feeding, successfully modeled obese mice were randomly allocated into the HFD group, the sham electroacupuncture group (HFD + sham) group, or the EA group.

### EA

EA group received a 4-week intervention. Mice were placed on a heating pad maintaining a body temperature fluctuating between 37 ± 0.5°Cwhile being anaesthetized with 2% isoflurane inhalation(RWD Life Science Co., Ltd, Shenzhen, China). Subsequently, a pair of unipolar stainless steel needles were obliquely inserted into the unilateral “ST25 (Tianshu)” acupoint (Standard Chinese Medicine Acupoint Number: ST25; Location: 5 mm lateral to the midline, at the junction of the upper two-thirds and lower one-third of the line connecting the xiphoid process and the upper border of the pubic symphysis). The needle handles were connected to a HANS electroacupuncture apparatus (Model LH402A, purchased from Beijing Huawei Technologies Co., Ltd.). The electroacupuncture stimulation parameters were as follows: current intensity 2.0 mA, pulse width 0.4 ms, frequency alternating between 2 Hz and 15 Hz every 3 seconds. Each stimulation session lasted 20 minutes. This procedure was performed 6 times per week for 4 weeks, alternating between the left and right ST25 acupoints (15). Mice were not restrained during EA. Mice in the control groups that did not receive EA intervention underwent the same anesthesia procedure. For the sham electroacupuncture group, mice were anesthetized, and then acupuncture needles were inserted unilaterally into the ST25 acupoint without being connected to the electroacupuncture apparatus. This procedure was performed 6 times per week for a total of 4 weeks.

### *In vivo* RAMP1 signaling blockade

Successfully modeled obese mice (a total of 39 mice) were intraperitoneally injected with either BIBN4096 (0.3 mg/kg, HY-10095, MCE) or vehicle (2% DMSO in saline) for 4 weeks. The BIBN4096 + EA group additionally received a 4-week electroacupuncture intervention. BIBN4096 is a potent and selective non-peptide antagonist of the CGRP1 receptor complex, consisting of CLR and RAMP1.

### CGRP 8–37 injection

Obese model mice (a total of 39 mice) were randomly assigned to the Solvent, CGRP 8-37, and CGRP 8-37+EA groups using a random number table. The solvent group received an intrathecal injection of normal saline (5μL), while the CGRP 8-37, and CGRP 8-37+EA groups received an intrathecal injection of CGRP 8-37 (5 nmol/5μL; CAS: 129121-73-9, Target Mol). The CGRP 8-37+EA group additionally underwent electroacupuncture intervention for four weeks.

### Colon-specific TRPV1 knock down

After the establishment of the obese mouse model, the mice (a total of 72mice) were randomly divided into eight groups. For viral injection, Scrambled-AAV-shRNA Control Virus (Applied Biological Materials Inc., AbMole, Cat. No. C273 (CS5011110); titer: 1.35×10¹² GC/mL) and TRPV1-AAV-shRNA Virus (Applied Biological Materials Inc., AbMole, Cat. No. C273 (CS5011110); titer: 7.76×10¹² GC/mL) were diluted to a concentration of 10¹² GC/mL and injected into both the proximal and distal colon (5 μL per region, administered as 1μL per site across 5 sites). The groups included: Scrambled shRNA group (injected with Scrambled-AAV-shRNA Control Virus), TRPV1 shRNA group (injected with TRPV1-AAV-shRNA Virus), TRPV1 shRNA+EA group (injected with TRPV1-AAV-shRNA Virus and received electroacupuncture at ST25), and TRPV1 shRNA+Sham group (injected with TRPV1-AAV-shRNA Virus and received sham electroacupuncture, in which needles were inserted into ST25 without electrical stimulation).

### Colon-specific ILC2 knock down

After the establishment of the obese mouse model, the mice (a total of 60 mice) were randomly divided into six groups. First, rAAV-NMUR1-CRE-WPRE (BrainVTA, titer: 5.65×10¹² vg/mL) was mixed separately with rAAV-EF1a-DIO-DTA-WPRE-hCH-poly (BrainVTA, titer: 5.98×10¹² vg/mL) and rAAV-EF1a-DIO-mCherry-WPRE-hCH-poly (BrainVTA, titer: 5.19×10¹² vg/mL) to generate two distinct viral mixtures. Each mixture was then injected into the proximal and distal colon (5 μL per region, administered as 1 μL per site across 5 sites per region).

### RTX injection

For targeted ablation of TRPV1-expressing nociceptors in the DRG, mice were (a total of 39 mice) anesthetized with 2% isoflurane inhalation. Using a 30G needle and a microsyringe, 10 μL of the capsaicin analog resiniferatoxin (2 ng/g, CAS No.: 57444-62-9, TargetMol) or vehicle solution (DMSO in saline) was administered via intrathecal injection at the L5-L6 spinal level (16). Ablation efficiency was confirmed by the hot plate test and by quantifying TRPV1-positive neurons in the DRG using immunofluorescence microscopy. After successful modeling, the obese mice were randomly divided into three groups using a random number table: the vehicle group received vehicle solution (DMSO in saline), the RTX group received the capsaicin analog resiniferatoxin, and the RTX+ EA group received resiniferatoxin combined with electroacupuncture treatment for 4 weeks.

### Serum lipid profile analysis

Following anesthesia, blood was collected from mice via the retro-orbital plexus. The blood samples were allowed to clot at room temperature for 1.5 hours, followed by centrifugation at 2,500×g for 15 minutes at 4 °C to separate the serum. Serum levels of high-density lipoprotein cholesterol (HDL-C, Kit No. A112-1-1, Nanjing Jiancheng Bioengineering Institute), low-density lipoprotein cholesterol (LDL-C, Kit No. A113-1-1, Nanjing Jiancheng Bioengineering Institute), free fatty acids (FFA, Kit No. A042-2-1, Nanjing Jiancheng Bioengineering Institute), triglycerides (TG, Kit No. A110-1-1, Nanjing Jiancheng Bioengineering Institute), total cholesterol (TC, Kit No. A111-1-1, Nanjing Jiancheng Bioengineering Institute), and glycerol (Kit No. F005-1-1, Nanjing Jiancheng Bioengineering Institute) were measured using commercial assay kits according to the manufacturers’ instructions. All measurements were analyzed using a Synergy HT microplate reader.

### Elisa

After homogenizing 30 mg of colon tissue using a ball mill homogenizer, the levels of IL-5 and IL-13 in the tissue were measured using Mouse IL-5 ELISA KIT (Cas:ml063157, Mlbio) and Mouse IL-13 ELISA KIT (Cas:ml106729, Mlbio), respectively. All procedures were performed strictly in accordance with the manufacturer’s instructions.

### RT-PCR

Total RNA was extracted from 30 mg of mouse colon tissue using the FastPure Complex Tissue/Cell Total RNA Isolation Kit (Cat. No. RC113-01, Vazyme). Subsequently, reverse transcription was performed using the All-In-One 5X RT MasterMix with gDNA Removal (Cat. No. G592, Applied Biological Materials). Finally, quantitative real-time PCR (qPCR) analysis was carried out using BlasTaq™ 2X qPCR MasterMix (Cat. No. G892, Applied Biological Materials). All procedures were conducted strictly in accordance with the manufacturer’s instructions. The primer sequences used for real-time PCR are as follows.

Mouse *GAPDH* (Forward):5′-GGTGCTGAGTATGTCGTGGAG-3′

Mouse *GAPDH* (reverse):5′-GTCTTCTGAGTGGCAGTGATG-3′

Mouse *Il-5* (forward): 5′-TGACAAGCAATGAGACGATGAGG-3′

Mouse *Il-5* (reverse): 5′-ACCCCCACGGACAGTTTGATTC-3′

Mouse *Il-13* (forward): 5′-CCTGGCTCTTGCTTGCCT-3′

Mouse *Il-13* (reverse): 5′-GGTCTTGTGTGATGTTGCTCA-3′

### Western blot

Proteins were extracted from harvested mouse colon tissues. Specifically, 30 mg of colon tissue was added to 300 μL of extraction buffer (a combination of protease inhibitor and RIPA buffer, Thermo Scientific Inc, Waltham). The tissue was homogenized using a bead mill homogenizer and then centrifuged at 12,000 rpm for 30 minutes. The supernatant was collected, and 30 μg of total tissue lysate was separated using SurePAGETM (4-20%, GenScript, China). Electrophoresis was performed at 150 V for 50 minutes. Proteins were transferred to a PVDF membrane using an e-Blot transfer system (GenScript, China). The membrane was blocked with 5% BSA for 1 hour at room temperature. Subsequently, the PVDF membrane was incubated overnight at 4 °C with the following primary antibodies: IL-13 (1:1000, Signalway Antibody, #38354), IL-5 (1:1000, Signalway Antibody, #41062), β-tubulin (1:6000, Proteintech Group, Cat NO.10094-1-AP) and β-actin(1:10000, Proteintech Group, Cat No. 20536-1-AP). After washing the primary antibodies three times for 5 minutes each with TBST, the membrane was incubated with the corresponding secondary antibody, Rabbit anti-Goat IgG HRP-conjugated (1:10000; Signalway Antibody, L3042), for 1 hour at room temperature. Finally, the membrane was washed three times for 5 minutes each with TBST to remove the secondary antibody.

### Hematoxylin and eosin staining

After euthanasia, the proximal and distal colon segments were excised and fixed in 4% paraformaldehyde for 24 hours. The tissues were then trimmed, embedded in paraffin, and sectioned into 5 μm thick slices. Staining was performed using a Hematoxylin and Eosin Staining Kit (Solarbio, Cat# G1121). All procedures were performed following the manufacturer’s instructions.

### Alcian blue-periodic acid schiff staining

The proximal and distal colon segments were fixed in 4% paraformaldehyde for at least 24 hours. The tissues were subsequently trimmed, embedded in paraffin, and sectioned into 5 μm thick slices. Staining was conducted using an Alcian Blue-Periodic Acid Schiff Staining Kit (Solarbio, Cat# G1285). All steps were carried out according to the manufacturer’s instructions.

### Immunofluorescence staining

Following cardiac perfusion, proximal and distal colon segments and DRG (T10-L1 for proximal colon innervation, L6-S1 for distal colon innervation) were collected and fixed overnight in 4% paraformaldehyde. Tissues were subsequently dehydrated in 30% sucrose solution for 24 hours at 4 °C. Colon tissues were embedded in optimal cutting temperature (OCT) compound and sectioned into 30 μm thick slices (colon) and 15 μm thick slices (DRG) using a cryostat (Leica, Germany). Sections were washed three times with PBS for 10 minutes each. Blocking was performed using Sea Block blocking buffer (Cat# 37527, Thermo Fisher Scientific, MA, USA) containing 0.3% Triton X-100 for 1 hour at 37 °C. The sections were then incubated with the following primary antibodies overnight at 4 °C: ST2 (1:200, Proteintech Group, Cat# 60112-1-Ig), CD90 (1:100, Invitrogen, REF: 25-9335-80), RAMP1 (1:300, Proteintech Group, Cat# 10327-1-AP), TRPV1 (1:500, Millipore Corporation, Cat# AB9554). After incubation, sections were washed three times with PBST (PBS containing 0.1% Tween-20) for 10 minutes each. Corresponding secondary antibodies were then applied: Donkey Anti-Mouse IgG H&L (Alexa Fluor^®^ 488) (1:500, AB150105), Donkey Anti-Rat IgG H&L (Alexa Fluor^®^ 594) (1:500, AB150156), Donkey Anti-Rabbit IgG H&L (Alexa Fluor^®^ 647) (1:500, AB10075), and Donkey Anti-Rabbit IgG H&L (Alexa Fluor^®^ 488) (1:500, AB150073), and incubated for 1 hour at 37 °C. Following secondary antibody incubation, sections were washed three times with PBST for 10 minutes each. Nuclei were stained with DAPI (CAS: C1006, Beyotime, Jiangsu, China) for 15 minutes at room temperature, followed by three 10-minute washes with PBS. Finally, sections were mounted using ProLong Diamond Antifade Mountant (Cat# P36990, Thermo Fisher Scientific), coverslipped, and the edges were sealed with nail polish. Mounted sections were allowed to cure for 36 hours at room temperature.

### Whole-mount colon TRPV1 and CGRP imaging

Following cardiac perfusion, the colon was placed in 4% paraformaldehyde and fixed overnight at 4 °C with gentle agitation. The 4% paraformaldehyde was then removed, and the tissue was washed three times for 2 hours each at room temperature with 0.01 M PBS on a shaker. The tissue was incubated with a delipidation solution for 24 hours, followed by three 2-hour washes with 0.01 M PBS at room temperature. Blocking was performed using Sea Block blocking buffer (REF: 37527, Thermo Fisher Scientific, MA, USA) containing 0.3% Triton X-100 for 1.5 hours. The tissue was then incubated with primary antibodies (TRPV1: 1:500, Millipore Corporation, Cat# AB9554; CGRP: 1:300, ABCAM, Cat# AB81887) for 6 days at 4 °C. After primary antibody incubation, the tissue was washed three times for 2 hours each with 0.01 M PBS at room temperature. Subsequently, the tissue was incubated with secondary antibodies Donkey Anti-Rabbit IgG H&L (Alexa Fluor^®^ 488) (1:500, AB150073) and Donkey Anti-Mouse IgG H&L (Alexa Fluor^®^ 594) (1:500, AB150110) for 4 days at 4 °C. Following secondary antibody incubation, the tissue was washed with 10% Triton X-100 in 0.01 M PBS for 15 minutes at room temperature, followed by three 2-hour washes with 0.01 M PBS at room temperature.

### Flow cytometry

Fresh colon tissues were collected, longitudinally opened, and washed. Tissues were cut into approximately 2 mm³ pieces and incubated in 10 mM EDTA/DMEM (Gibco, REF: C11995500BT) at 37 °C with shaking at 80 rpm for 20 minutes. Tissue fragments were then transferred to DPBS, vortexed four times, and the supernatant containing intestinal epithelial cells was discarded. The remaining tissue was minced and further digested in 5 mL of DMEM containing Liberase TL Research Grade (REF: 05401020001) and 1 U/mL DNase I (BioFROX, CAS NO.: 9003-98-9) at 37 °C with shaking at 80 rpm for 40 minutes. The resulting cell suspension was filtered through a 100 μm strainer. Cells were centrifuged at 500 × g for 5 minutes at 4 °C and washed twice with DPBS. Non-specific binding was blocked using CD16/CD32 (0.5 μL/test, Invitrogen, REF: 14-0161-81) on ice for 10 minutes. Surface antibodies were then added for staining on ice for 30 minutes: CD45 (0.625 μL/test, Invitrogen, REF: 47-0451-80), Hematopoietic Lineage (20 μL/test, Invitrogen, REF: 22-7770-72), CD90.2 (0.12 μL/test, Invitrogen, REF: 12-0902-81), and ST2 (0.15 μL/test, Invitrogen, REF: 25-9335-80). After staining, cells were centrifuged at 500 × g for 5 minutes at 4 °C, the supernatant was discarded, and the cells were washed twice with DPBS. Finally, the cells were filtered and analyzed using a CytoFLEX flow cytometer with CytExpert software.

### Experimental design

This study was designed to evaluate the therapeutic effect of electroacupuncture on established obesity. Therefore, obesity was first induced by a high-fat diet, and mice that successfully developed obesity were subsequently selected for the treatment phase. During the treatment phase, all obese mice were randomly assigned to either the control group or the intervention group, and both groups continued to receive the high-fat diet to ensure identical dietary conditions, thereby allowing the effect of the intervention itself to be evaluated. To minimize bias, this study incorporated randomization and blinding throughout the experimental process. Animals were randomly assigned to experimental groups using a random number table. The investigators responsible for treatment administration and subsequent data collection were blinded to the group allocation. For outcome assessment, histological scoring and Western blot band densitometry were conducted by at least two independent examiners who were unaware of the experimental group identities.

### Data analysis

All statistical analyses were performed using IBM SPSS Statistics 26 and GraphPad Prism 8 software. Data are presented as mean ± standard error of the mean (SEM). All data met the assumption of normal distribution. Comparisons between two groups were analyzed using the independent samples t-test. For comparisons involving more than two groups or two factors, one-way ANOVA and two-way ANOVA were employed, respectively. Following one-way ANOVA, Tukey’s HSD *post hoc* test was conducted for multiple comparisons. Following two-way ANOVA, Sidak’s multiple comparisons test was performed to strictly control for false positives. A p-value ≤ 0.05 was considered statistically significant, while “ns” indicates no significant difference.

## Results

### Electroacupuncture ameliorates metabolic disorders and activates type 2 immunity in the colon to ameliorate HFD-induced intestinal injury

To evaluate the effect of EA on metabolism in obese mice, we administered a 4-week EA intervention to successfully modeled obese mice induced by a high-fat diet. The results showed that compared with the ND group, high-fat diet feeding significantly increased mouse body weight (**Figure 1A**), whereas EA significantly reduced both body weight and food intake in obese mice (**Figures 1B, C**). Under HFD conditions, EA reduced the wet weights of both inguinal white adipose tissue (iWAT) and epididymal white adipose tissue (eWAT) (**Supplementary Figures S1A, B**). To further assess the regulatory effect of EA on serum lipid metabolism in obese mice, the results demonstrated that compared to the ND group, serum levels of NEFA, TG, T-CHO, LDL-C, and Glycerol were significantly increased, while HDL-C levels were decreased in the HFD group. EA treatment significantly downregulated serum levels of NEFA, TG, T-CHO, LDL-C, and Glycerol, and increased HDL-C levels in obese mice (**Supplementary Figures S1C–H**). These results suggest that EA may improve lipid profiles in HFD-fed obese mice; however, because EA also reduced food intake, these metabolic improvements may be partly secondary to decreased energy intake rather than solely reflecting a direct effect on lipid metabolism.

**Figure 1:**
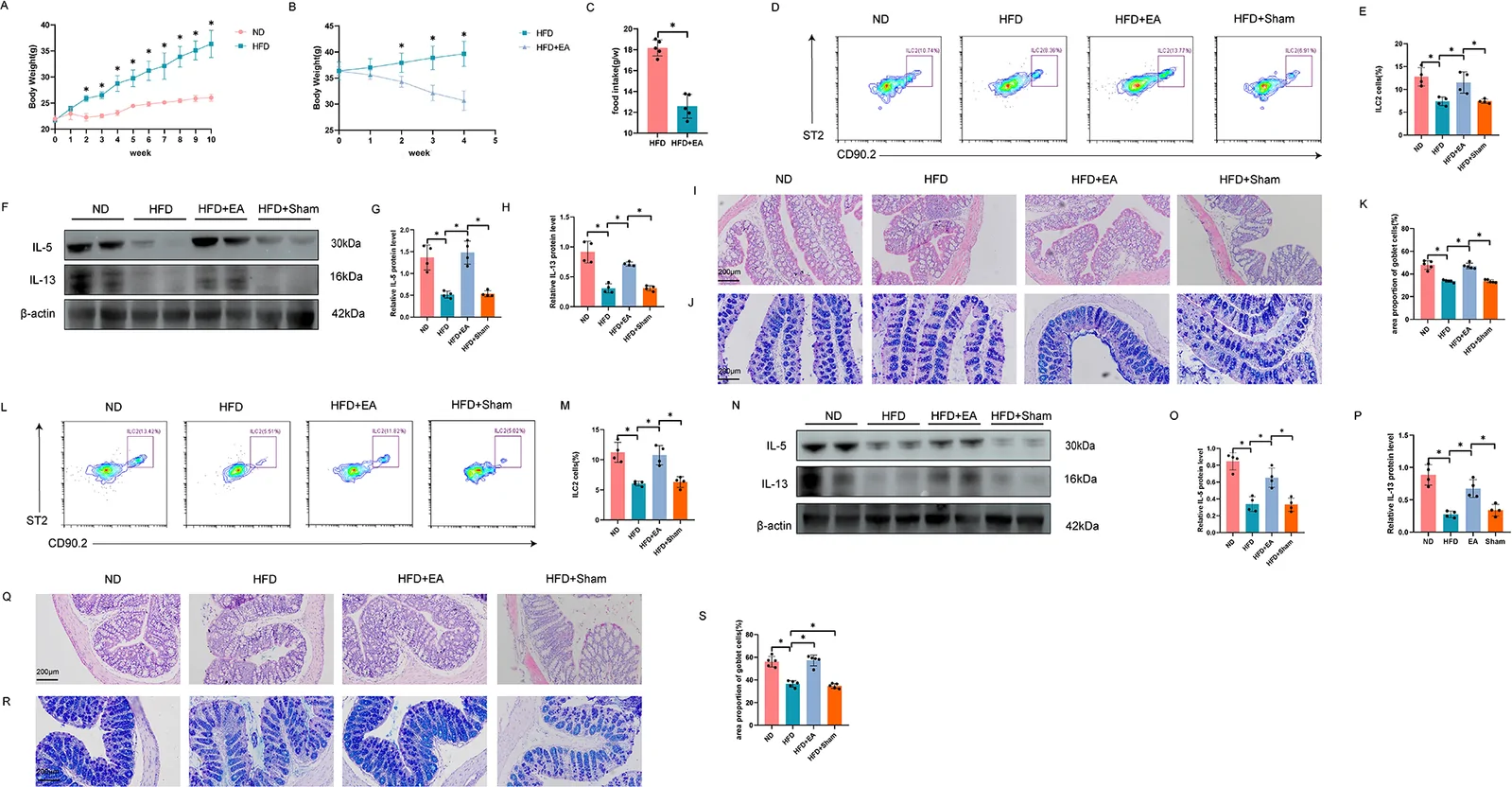
Electroacupuncture promotes intestinal type 2 immunity and repairs intestinal injury. **(A)** Body weight of mice after 10 weeks of feeding. **(B)** Body weight of mice in the HFD and EA groups. **(C)** Food intake of mice in the HFD and EA groups. **(D, E)** Representative flow cytometry plots and quantification of ILC2s in the proximal colon of ND, HFD, HFD + EA and HFD + Sham groups. **(F–H)** Western blot analysis and quantification of IL-5 and IL-13 protein levels in the proximal colon of ND, HFD, HFD + EA and HFD + Sham groups. **(I)** Representative H&E staining images of the proximal colon in ND, HFD, HFD + EA and HFD + Sham groups. **(J, K)** Representative AB-PAS staining images and quantification of goblet cell ratio in the proximal colon of ND, HFD, HFD + EA and HFD + Sham groups. **(L, M)** Representative flow cytometry plots and quantification of ILC2s in the distal colon of ND, HFD, HFD + EA and HFD + Sham groups. **(K–P)** Western blot analysis and quantification of IL-5 and IL-13 protein levels in the distal colon of ND, HFD, HFD + EA and HFD + Sham groups. **(Q)** Representative H&E staining images of the distal colon in ND, HFD, HFD + EA and HFD + Sham groups. **(R, S)** Representative AB-PAS staining images and quantification of goblet cell ratio in the distal colon of ND, HFD, HFD + EA and HFD + Sham groups. **(A–C, I–K, Q–S)**: n=5; **(D–H, L–P)** :n=4. **(I–J, Q, R)**: Scale bar = 200 µm. Error bars represent mean ± SEM. Statistical significance was determined by two-way ANOVA (Sidak’s multiple comparisons test), one-way ANOVA (Tukey’s HSD *post hoc* test) and independent samples t-test. ns indicates no significant difference; *p < 0.05.

To investigate whether EA activates colonic mucosal type 2 immunity under obese conditions, we assessed ILC2 cells and their secreted factors. Flow cytometry data revealed that HFD feeding decreased the abundance of colonic ILC2s, while EA treatment upregulated ILC2 levels (Proximal colon: **Figures 1D, E**; Distal colon: **Figures 1L, M**). To further validate the flow cytometry results, we evaluated the expression of type 2 cytokines secreted by ILC2s using Western blot. The results showed that compared to the ND group, the HFD group exhibited reduced secretion of IL-5 and IL-13 (Proximal colon: **Figures 1F–H**; Distal colon: **Figures 1N–P**), which was reversed by EA treatment. However, the HFD + sham group did not exhibit elevated levels of colonic ILC2 cells or type 2 inflammatory cytokines compared with the HFD group. These results suggest that EA activates colonic mucosal type 2 immunity in obesity. Since the activation of intestinal mucosal type 2 immunity can promote goblet cell generation and drive intestinal barrier remodeling, we further evaluated whether EA could modulate HFD-induced mucosal injury. Histopathological sections showed that compared to the ND group, the HFD group exhibited disorganized glandular architecture and a significant reduction in goblet cell numbers. EA intervention significantly increased goblet cell numbers in both proximal and distal colon (Proximal colon: **Figures 1I–K**; Distal colon: **Figures 1Q–S**) and ameliorated intestinal injury. Compared with the HFD group, the HFD + sham group showed no significant changes in colonic histological sections.

### RAMP1 expression is required for EA-mediated activation of intestinal type 2 immunity in obese mice

ILC2s express various neuropeptide receptors, among which RAMP1, a receptor component for CGRP, is highly expressed on their surface. We hypothesized that EA activates intestinal mucosal type 2 immunity and repairs intestinal injury by modulating RAMP1 expression on ILC2s. To test this hypothesis, we first investigated whether EA regulates RAMP1-positive ILC2s. Results showed that HFD feeding decreased the number of RAMP1-positive ILC2 cells compared to the ND group, and EA upregulated its expression. In addition, no statistically significant difference was observed between the HFD + sham group and the HFD group (Proximal colon: **Figures 2A, B**; Distal colon: **Figures 2C, D**). Subsequently, we ablated RAMP1 on colonic ILC2s by intraperitoneal injection of the RAMP1 antagonist BIBN4096. Flow cytometry results showed that compared to the HFD + Vehicle group, both the BIBN4096 and BIBN4096+EA groups exhibited significantly downregulated ILC2 abundance in the proximal and distal colon, with no statistical difference between these two groups (Proximal colon: **Figures 2E, F**; Distal colon: **Figures 2O, P**). To further confirm this, immunofluorescence staining for ILC2s (marked by ST2 and CD90) showed that BIBN4096 treatment significantly reduced ILC2 abundance (Proximal colon: **Figures 2G, H**; Distal colon: **Figures 2Q, R**) and the levels of secreted type 2 cytokines IL-5 and IL-13, with no significant difference between the BIBN4096 and BIBN4096+EA groups (Proximal colon: **Figures 2I–K**; Distal colon: **Figures 2S–U**). This indicates that RAMP1 ablation suppresses intestinal type 2 immunity and inhibits the efficacy of EA. To assess the impact of EA on the intestinal barrier under RAMP1 ablation, we evaluated intestinal pathology. Results showed that compared to the HFD + Vehicle group, BIBN4096-treated mice exhibited disorganized glands and a significant decrease in goblet cell numbers in both proximal and distal colon (Proximal colon: **Figures 2L–N**; Distal colon: **Figures 2V–X**), indicating more severe pathology and a loss of the restorative effect of EA. These data collectively demonstrate that RAMP1 expression is essential for EA to activate intestinal type 2 immunity and repair intestinal injury in obese mice. Loss of RAMP1 signaling impairs this tissue-protective type 2 immune response. To corroborate the role of CGRP as a key mediator, a more direct loss-of-function experiment was conducted by intrathecally injecting the CGRP receptor antagonist CGRP 8-37. The results showed a marked decrease in ILC2 cell numbers (Proximal colon: **Figures 2Y, Z**; Distal colon: **Figures 2Kk, Ll**), as well as downregulated levels of the type 2 immune cytokines IL-5 and IL-13 secreted by these cells (Proximal colon: **Figures 2Aa–Gg**; Distal colon: **Figures 2Mm–Ss**), as demonstrated by Western blotting, RT-PCR, and ELISA. Histological analysis revealed that mice treated with CGRP 8–37 exhibited significant atrophy of colonic crypts and a reduced number of goblet cells (Proximal colon: **Figures 2Hh–Jj**; Distal colon: **Figures 2Tt–Vv**). Under these conditions, the effects of electroacupuncture were suppressed.

**Figure 2:**
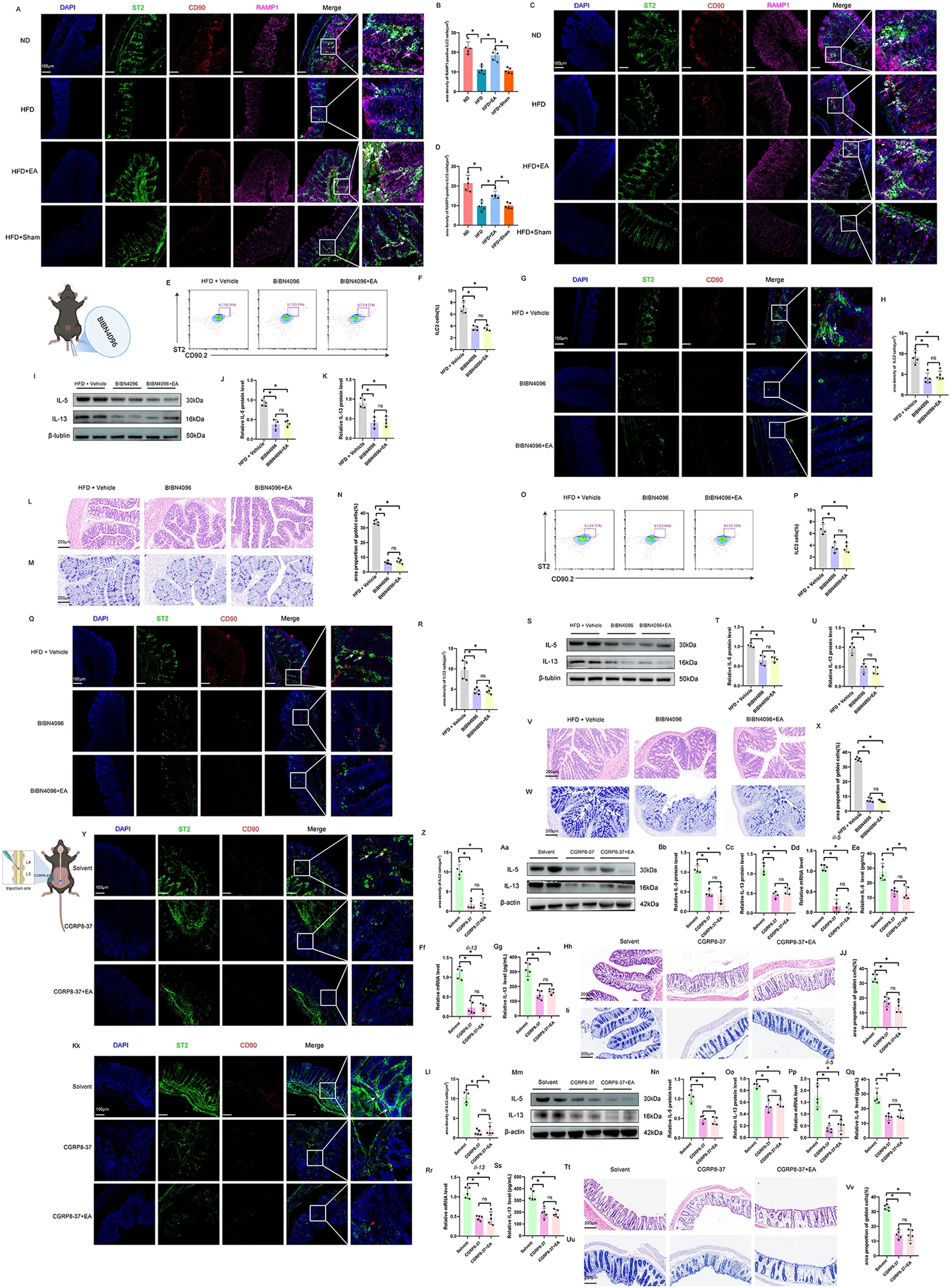
Electroacupuncture mediates activation of intestinal type 2 immunity in a RAMP1-dependent manner. **(A)** Representative immunofluorescence images of DAPI (blue), ST2 (green), CD90 (red), and RAMP1 (purple) in the proximal colon of ND, HFD, HFD + EA and HFD + Sham groups. **(B)** Percentage of RAMP1-positive ILC2s in the proximal colon of ND, HFD, HFD + EA and HFD + Sham groups. **(C)** Representative immunofluorescence images of DAPI (blue), ST2 (green), CD90 (red), and RAMP1 (purple) in the distal colon of ND, HFD, HFD + EA and HFD + Sham groups. **(D)** Percentage of RAMP1-positive ILC2s in the distal colon of ND, HFD, HFD + EA and HFD + Sham groups. **(E, F)** Representative flow cytometry plots and quantification of ILC2s in the proximal colon of HFD + Vehicle, BIBN4096, and BIBN4096+EA groups. **(G)** Representative immunofluorescence images of DAPI (blue), ST2 (green), and CD90 (red) in the proximal colon of HFD + Vehicle, BIBN4096, and BIBN4096+EA groups. **(H)** Percentage of ILC2s in the proximal colon of Ctrl, BIBN4096, and BIBN4096+EA groups. **(I–K)** Western blot analysis and quantification of IL-5 and IL-13 protein levels in the proximal colon of HFD + Vehicle, BIBN4096, and BIBN4096+EA groups. **(L)** Representative H&E staining images of the proximal colon in Ctrl, BIBN4096, and BIBN4096+EA groups. **(M, N)** Representative AB-PAS staining images and quantification of goblet cell ratio in the proximal colon of HFD + Vehicle, BIBN4096, and BIBN4096+EA groups. **(O, P)** Representative flow cytometry plots and quantification of ILC2s in the distal colon of HFD + Vehicle, BIBN4096, and BIBN4096+EA groups. **(Q)** Representative immunofluorescence images of DAPI (blue), ST2 (green), and CD90 (red) in the distal colon of Ctrl, BIBN4096, and BIBN4096+EA groups. **(R)** Percentage of ILC2s in the distal colon of HFD + Vehicle, BIBN4096, and BIBN4096+EA groups. **(S–U)** Western blot analysis and quantification of IL-5 and IL-13 protein levels in the distal colon of HFD + Vehicle, BIBN4096, and BIBN4096+EA groups. **(V)** Representative H&E staining images of the distal colon in HFD + Vehicle, BIBN4096, and BIBN4096+EA groups. **(W, X)** Representative AB-PAS staining images and quantification of goblet cell ratio in the distal colon of HFD + Vehicle, BIBN4096, and BIBN4096+EA groups. **(Y)** Representative immunofluorescence images of DAPI (blue), ST2 (green), and CD90 (red) in the proximal colon of solvent, CGRP 8-37, and CGRP 8-37+EA groups. **(Z)** Percentage of ILC2s in the proximal colon of solvent, CGRP 8-37, and CGRP 8-37+EA groups. **(Aa–Cc)** Western blot analysis and quantification of IL-5 and IL-13 protein levels in the proximal colon of Solvent, CGRP 8-37, and CGRP 8-37+EA groups. **(Dd)** Relative *il-5* mRNA expression in the proximal colon of Solvent, CGRP 8-37, and CGRP 8-37 +EA groups. **(Ee)** Relative IL-5 expression in the proximal colon of Solvent, CGRP 8-37, and CGRP 8-37+EA groups. **(Ff)** Relative *il-13* mRNA expression in the proximal colon of Solvent, CGRP 8-37, and CGRP 8-37+EA groups. **(Gg)** Relative IL-13 expression in the proximal colon of Solvent, CGRP 8-37, and CGRP 8-37 +EA groups. (Hh) Representative H&E staining images of the proximal colon in Solvent, CGRP 8-37, and CGRP 8-37+EA groups. **(Jj–Ll)** Representative AB-PAS staining images and quantification of goblet cell ratio in the proximal colon of Solvent, CGRP 8-37, and CGRP 8-37+EA groups. **(Kk)** Representative immunofluorescence images of DAPI (blue), ST2 (green), and CD90 (red) in the distal colon of Solvent, CGRP 8-37, and CGRP 8-37+EA groups. **(Ll)** Percentage of ILC2s in the distal colon of Solvent, CGRP 8-37, and CGRP 8-37+EA groups. **(Mm–Oo)** Western blot analysis and quantification of IL-5 and IL-13 protein levels in the distal colon of Solvent, CGRP 8-37, and CGRP 8-37+EA groups. **(Pp)** Relative *il-5* mRNA expression in the distal colon of Solvent, CGRP 8-37, and CGRP 8-37+EA groups. **(Qq)** Relative IL-5 expression in the distal colon of Solvent, CGRP 8-37, and CGRP 8-37+EA groups. **(Rr)** Relative *il-13* mRNA expression in the distal colon of Solvent, CGRP 8-37, and CGRP 8-37+EA groups. **(Ss)** Relative IL-13 expression in the distal colon of Solvent, CGRP 8-37, and CGRP 8-37+EA groups. **(Tt)** Representative H&E staining images of the distal colon in Solvent, CGRP 8-37, and CGRP 8-37+EA groups. **(Uu, Vv)** Representative AB-PAS staining images and quantification of goblet cell ratio in the distal colon of Solvent, CGRP 8-37, and CGRP 8-37+EA groups. **(A–D, G–N, Q, R, V–Z, Dd–Ll, Pp–Vv)** n=5. **(E, F, I–K, O, P, S–U, Aa–Cc, Mm–Oo)** n=4. **(A, C, G, Q, Y, Kk)** Scale bar = 100 µm; **(L, M, V, W, Hh, li, Tt, Uu)** Scale bar = 200 µm. Error bars represent mean ± SEM. Data were analyzed by one-way ANOVA (Tukey’s HSD *post hoc* test). ns indicates no significant difference; *p < 0.05.

### EA promotes intestinal mucosal type 2 immunity via the TRPV1-CGRP-RAMP1 axis in obese mice

Our data suggest that EA activates intestinal type 2 immunity by modulating the expression of RAMP1, the co-receptor for CGRP, on ILC2s. Immunofluorescence revealed co-localization of CGRP with TRPV1 in colonic nerves, suggesting that CGRP released from TRPV1 positive sensory nerves is a likely source. We therefore hypothesized that EA signals through TRPV1 derived CGRP acting on RAMP1 on ILC2s to promote type 2 immunity. We detected TRPV1 derived CGRP expression in proximal and distal colon. TRPV1 was distributed and expressed in both the myenteric plexus and crypts, a pattern also supported by Zhang’s research (9). Results showed that compared with the ND group, both the HFD group and the sham electroacupuncture group exhibited reduced length of CGRP derived from TRPV1-expressing sensory nerve fibers in the myenteric plexus and crypts of the proximal and distal colon, whereas electroacupuncture intervention increased their expression. (Proximal colon: Myenteric plexus: **Figures 3A, E**; Crypts: **Figure 4B**. Distal colon: Myenteric plexus: **Figures 3C, F**; Crypts: **Figure 3D**). To determine the location of dorsal root ganglion (DRG) cell bodies in extrinsic enteric sensory neurons, we injected the fluorescent retrograde tracer cholera toxin subunit B (CTB) into the wall of different intestinal segments and dissected the DRGs projecting to the intestine. Individual CTB tracing of the proximal and distal intestine revealed that CTB injection into the proximal colon retrogradely labeled DRG segments from T10 to L1 (**Figure 3G**), whereas CTB injection into the distal colon retrogradely labeled DRG segments from L6 to S1 (**Figure 3L**). We also examined TRPV1 neuron expression in the DRG. Results showed that compared with the ND group, both the HFD group and the sham electroacupuncture group exhibited reduced TRPV1^+^ neuron abundance, whereas electroacupuncture intervention upregulated it. (Innervating proximal colon: **Figures 3H, I**; Innervating distal colon: **Figures 3M, N**). Furthermore, we measured the expression of the neuronal activity marker cFos in relevant DRG neurons shortly after EA. The results showed that the number of CGRP and cFos double-labeled neurons in the DRG was significantly reduced in the HFD group. EA intervention markedly increased the number of CGRP and cFos co-expressing neurons, indicating that EA activates CGRP-expressing neurons in the DRG. No statistically significant difference was observed between the HFD + sham group and the HFD group (Proximal colon: **Figures 3J, K**; Distal colon: **Figures 3O, P**). These findings provide more direct functional evidence that EA at ST25 directly activates the TRPV1-CGRP cascade.

**Figure 3:**
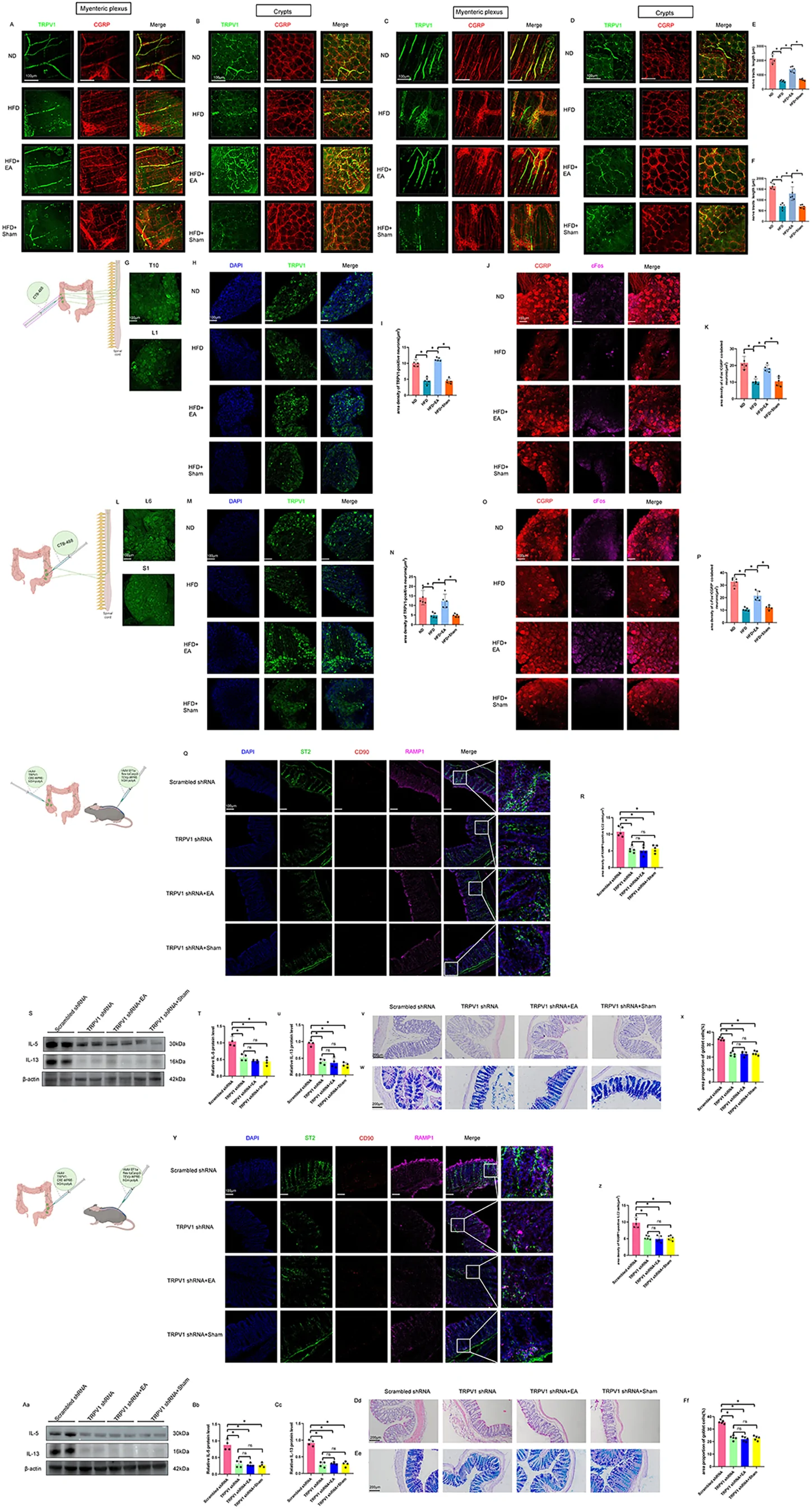
Electroacupuncture activates the expression of TRPV1+ neurons in the colon and DRG. **(A, E)** Representative immunofluorescence images of TRPV1 (green) and CGRP (red), and quantification of TRPV1/CGRP co-localized nerve tract length in the myenteric plexus of the proximal colon in ND, HFD, HFD+ EA and HFD+ Sham groups. **(B)** Representative immunofluorescence images of TRPV1 (green) and CGRP (red) in the crypts of the proximal colon in ND, HFD, HFD+ EA and HFD+ Sham groups. **(C, F)** Representative immunofluorescence images of TRPV1 (green) and CGRP (red), and quantification of TRPV1/CGRP co-localized nerve tract length in the myenteric plexus of the distal colon in ND, HFD, HFD+ EA and HFD + Sham groups. **(D)** Representative immunofluorescence images of TRPV1 (green) and CGRP (red) in the crypts of the distal colon in ND, HFD, HFD+ EA and HFD + Sham groups. **(G)** Representative immunofluorescence images of CTB (green) in the T10 and L1 DRG. **(H)** Representative immunofluorescence images of DAPI (blue) and TRPV1 (green) in the T10-L1 DRG of ND, HFD, HFD+ EA and HFD+ Sham groups. **(I)** The area density of TRPV1-positive neurons. **(J)** Representative immunofluorescence images of CGRP (red) and c-Fos (purple) in the T10-L1 DRG of ND, HFD, HFD+ EA and HFD+ Sham groups. **(K)** The area density of CGRP^+^/c-Fos^+^ co-labeled neurons. **(L)** Representative immunofluorescence images of CTB (green) in the L6-S1 DRG. **(M)** Representative immunofluorescence images of DAPI (blue) and TRPV1 (green) in the L6-S1 DRG of ND, HFD, HFD+ EA and HFD+ Sham groups. **(N)** The area density of TRPV1-positive neurons. **(O)** Representative immunofluorescence images of CGRP (red) and c-Fos (purple) in the L6-S1 DRG of ND, HFD, HFD+ EA and HFD+ Sham groups. **(P)** The area density of CGRP^+^/c-Fos^+^ co-labeled neurons. **(Q)** Representative immunofluorescence images of DAPI (blue), ST2 (green), CD90 (red), and RAMP1 (purple) in the proximal colon of Scrambled shRNA, TRPV1 shRNA, TRPV1 shRNA + EA and TRPV1 shRNA +Sham groups. **(R)** Percentage of RAMP1-positive ILC2s in the proximal colon of Scrambled shRNA, TRPV1 shRNA, TRPV1 shRNA + EA and TRPV1 shRNA +Sham groups. **(S–U)** Western blot analysis and quantification of IL-5 and IL-13 protein levels in the proximal colon of Scrambled shRNA, TRPV1 shRNA, TRPV1 shRNA + EA and TRPV1 shRNA +Sham groups. **(V)** Representative H&E staining images of the proximal colon in Scrambled shRNA, TRPV1 shRNA, TRPV1 shRNA+ EA and TRPV1 shRNA+ Sham groups. **(W,X)** Representative AB-PAS staining images and quantification of goblet cell ratio in the proximal colon of Scrambled shRNA, TRPV1 shRNA, TRPV1 shRNA+ EA and TRPV1 shRNA +Sham groups. **(Y)** Representative immunofluorescence images of DAPI (blue), ST2 (green), CD90 (red), and RAMP1 (purple) in the distal colon of Scrambled shRNA, TRPV1 shRNA, TRPV1 shRNA + EA and TRPV1 shRNA +Sham groups. **(Z)** Percentage of RAMP1-positive ILC2s in the distal colon of Scrambled shRNA, TRPV1 shRNA, TRPV1 shRNA+ EA and TRPV1 shRNA +Sham groups. **(Aa–Cc)** Western blot analysis and quantification of IL-5 and IL-13 protein levels in the distal colon of Scrambled shRNA, TRPV1 shRNA, TRPV1 shRNA+ EA and TRPV1 shRNA+ Sham groups. **(Dd)** Representative H&E staining images of the distal colon in Scrambled shRNA, TRPV1 shRNA, TRPV1 shRNA+ EA and TRPV1 shRNA +Sham groups. **(Ee, Ff)** Representative AB-PAS staining images and quantification of goblet cell ratio in the distal colon of Scrambled shRNA, TRPV1 shRNA, TRPV1 shRNA+ EA and TRPV1 shRNA +Sham groups. **(A–R, V, X, Y, Z, Dd–Ff)** n=5; **(S–U, Aa–Cc)** n=4. **(A–Q)**, Y: Scale bar = 100 µm; **(V-W, Dd–Ee)** Scale bar = 200 µm. Error bars represent mean ± SEM. Data were analyzed by one-way ANOVA (Tukey’s HSD *post hoc* test). ns indicates no significant difference; *p < 0.05.

**Figure 4:**
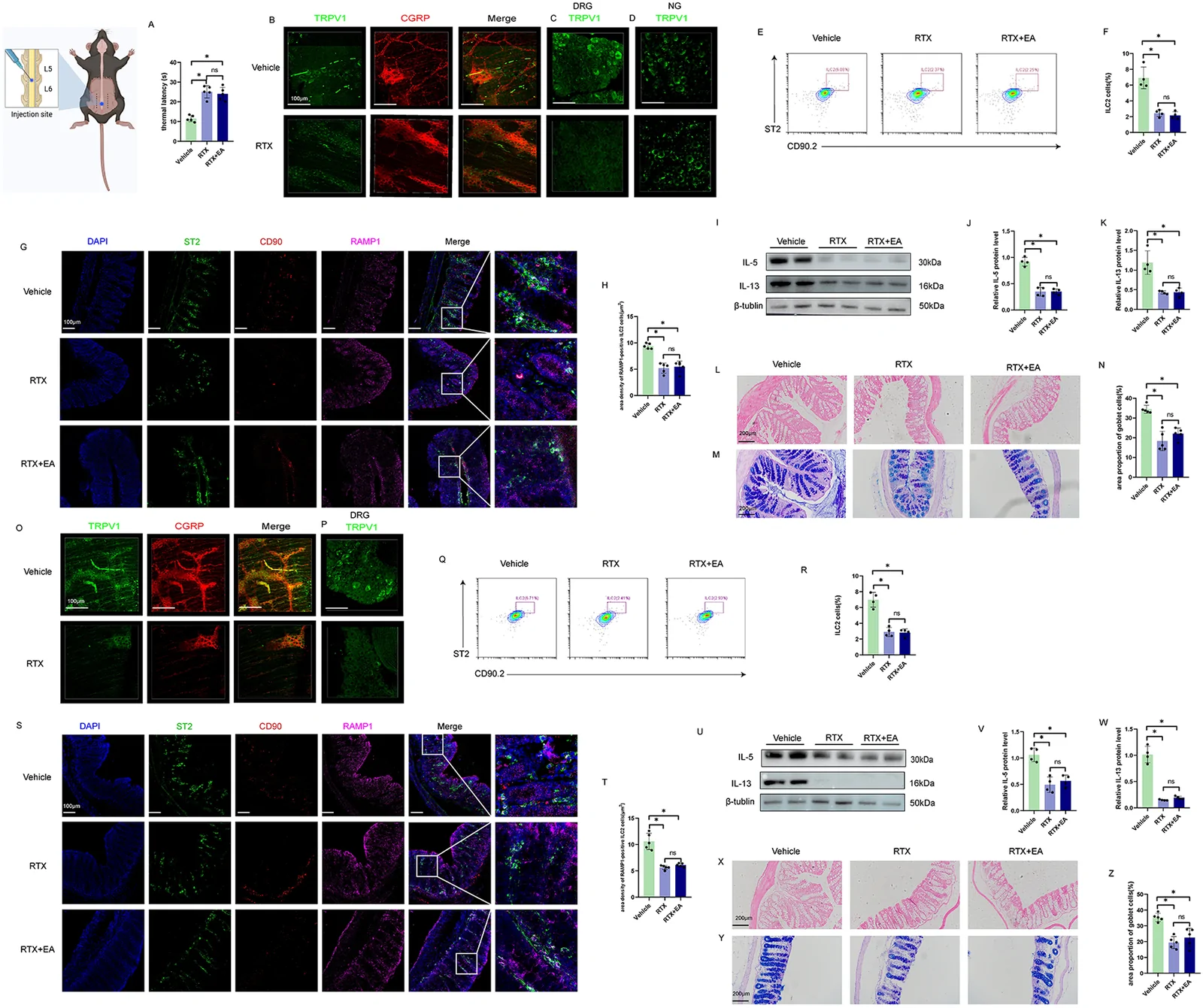
TRPV1^+^ DRG nociceptors are the key neuronal subset mediating electroacupuncture-induced repair of HFD-induced intestinal injury. **(A)** Latency in the hot plate test (55 °C) for Vehicle and RTX groups. **(B)** Representative immunofluorescence images of TRPV1 (green) and CGRP (red) in the proximal colon of Vehicle and RTX groups. **(C)** Representative immunofluorescence images of TRPV1^+^ neurons (green) in the T10-S1 DRG of Vehicle and RTX groups. **(D)** Representative immunofluorescence images of TRPV1^+^ neurons (green) in the NG of Vehicle and RTX groups. **(E, F)** Representative flow cytometry plots and quantification of ILC2s in the proximal colon of Vehicle, RTX, and RTX+EA groups. **(G)** Representative immunofluorescence images of DAPI (blue), ST2 (green), CD90 (red), and RAMP1 (purple) in the proximal colon of Vehicle, RTX, and RTX+EA groups. **(H)** Percentage of RAMP1-positive ILC2s in the proximal colon of Vehicle, RTX, and RTX+EA groups. **(I–K)** Western blot analysis and quantification of IL-5 and IL-13 protein levels in the proximal colon of Vehicle, RTX, and RTX+EA groups. **(L)** Representative H&E staining images of the proximal colon in Vehicle, RTX, and RTX+EA groups. **(M, N)** Representative AB-PAS staining images and quantification of goblet cell ratio in the proximal colon of Vehicle, RTX, and RTX+EA groups. **(O)** Representative immunofluorescence images of TRPV1 (green) and CGRP (red) in the distal colon of Vehicle and RTX groups. **(P)** Representative immunofluorescence images of TRPV1+ neurons (green) in the L6-S1 DRG of Vehicle and RTX groups. **(Q, R)** Representative flow cytometry plots and quantification of ILC2s in the distal colon of Vehicle, RTX, and RTX+EA groups. **(S)** Representative immunofluorescence images of DAPI (blue), ST2 (green), CD90 (red), and RAMP1 (purple) in the distal colon of Vehicle, RTX, and RTX+EA groups. **(T)** Percentage of RAMP1-positive ILC2s in the distal colon of Vehicle, RTX, and RTX+EA groups. **(U–W)** Western blot analysis and quantification of IL-5 and IL-13 protein levels in the distal colon of Vehicle, RTX, and RTX+EA groups. **(X)** Representative H&E staining images of the distal colon in Vehicle, RTX, and RTX+EA groups. **(Y, Z)** Representative AB-PAS staining images and quantification of goblet cell ratio in the distal colon of Vehicle, RTX, and RTX+EA groups. **(A–D, G, H, L–N, O, P, S, T, X, Z)** n=5; **(E, F, I–K, Q, R, U–W)** n=4. **(A–D, G, O, P, S)** Scale bar = 100 µm; **(L, M, X, Y)** Scale bar =200 µm. Error bars represent mean ± SEM. Data were analyzed by one-way ANOVA (Tukey’s HSD *post hoc* test). ns indicates no significant difference; *p < 0.05.

To further investigate whether electroacupuncture promotes the type 2 immune response in the colonic mucosa by activating the TRPV1–CGRP axis and its subsequent binding to RAMP1 on ILC2 cells, we performed targeted ablation of TRPV1-expressing neurons in the colon via site-specific injections of TRPV1-AAV-shRNA Virus into the proximal and distal colonic walls. Compared with mice injected with Scrambled-AAV-shRNA Control Virus, those receiving TRPV1-AAV-shRNA Virus exhibited marked loss of TRPV1^+^ and CGRP^+^ nerve fibers in the colon (proximal colon: **Supplementary Figure S2C**; distal colon: **Supplementary Figure S2D**). We next examined the expression of RAMP1-positive ILC2 cells and type 2 inflammatory cytokines. The results demonstrated that, relative to the Scrambled shRNA group, intervention with TRPV1-AAV-shRNA Virus significantly reduced the levels of RAMP1-positive ILC2 cells as well as IL-5 and IL-13 expression across all three groups. Moreover, under conditions of TRPV1 ablation, electroacupuncture failed to restore their expression (proximal colon: **Figures 3Q–U**; distal colon: **Figures 3Y–Cc**). These findings indicate that TRPV1 ablation leads to a marked suppression of the type 2 immune response in the colonic mucosa and abrogates the therapeutic effect of electroacupuncture. Histological examination of colonic sections further revealed that mice administered TRPV1-AAV-shRNA Virus displayed exacerbated pathological damage, characterized by loosened glandular arrangement and reduced mucin content (proximal colon: **Figures 3V–X**; distal colon: **Figures 3Dd–Ff**). Collectively, these results suggest tha**t** EA likely activates intestinal type 2 immunity via CGRP derived from TRPV1-expressing sensory nerve fibers binding to RAMP1 on ILC2s.

### TRPV1^+^ DRG nociceptors are key neurons mediating EA-induced intestinal type 2 immunity and repair in obese mice

We next sought to identify the key neuronal subset mediating this process. Previous studies indicate that Trpv1 mRNA is highly enriched in DRG but not expressed in enteric neurons (17). Therefore, our targeted RTX injection specifically ablates extrinsic TRPV1^+^ neurons without affecting enteric neurons.

To further investigate the role of DRG derived TRPV1-expressing neurons in intestinal repair via type 2 immunity, we ablated TRPV1^+^ DRG neurons by intrathecal RTX injection. Our results confirmed that RTX treatment reduced the latency to response in the hot plate test (**Figure 4A**), resulted in loss of extrinsic TRPV1^+^/CGRP^+^ nerve fibers projecting into the myenteric plexus (Proximal colon: **Figure 4B**; Distal colon: **Figure 4O**), and significantly decreased TRPV1^+^ neurons in the DRG (Innervating proximal colon: **Figure 4C**; Innervating distal colon: **Figure 4P**), while vagal TRPV1+ neurons were unaffected (**Figure 4D**). Under HFD conditions, ablation of TRPV1^+^ DRG neurons significantly decreased ILC2 abundance, with no significant difference between the RTX and RTX+EA groups (Proximal colon: **Figures 4E, F**; Distal colon: **Figures 4Q, R**). To determine whether EA acts through TRPV1^+^ DRG neurons to mediate the CGRP-RAMP1 signaling in ILC2s, we assessed RAMP1 expression on ILC2s and the levels of IL-5 and IL-13 by immunofluorescence and Western blot. Results showed that compared to the vehicle group, RTX-treated obese mice exhibited decreased RAMP1-positive ILC2s (Proximal colon: **Figures 4G, H**; Distal colon: **Figures 4S, T**) and reduced IL-5 and IL-13 levels (Proximal colon: **Figures 4I–K**; Distal colon: **Figures 4U–W**), with no difference between RTX and RTX+EA groups. This indicates that EA promotes intestinal type 2 immunity via TRPV1^+^ DRG-mediated CGRP-RAMP1 signaling on ILC2s. Colonic pathological sections showed that TRPV1^+^ DRG-ablated obese mice exhibited more severe pathology, characterized by disorganized glands, crypt atrophy, and significantly reduced goblet cell numbers in both RTX and RTX+EA groups compared to the vehicle group (Proximal colon: **Figures 4L–N**; Distal colon: **Figures 4X–Z**). These results collectively indicate that extrinsic TRPV1^+^ DRG neurons, but not vagal neurons, are the key neuronal subpopulation mediating EA-induced intestinal type 2 immunity and repair of intestinal injury in obese mice.

### Ablation of intestinal ILC2s abolishes the protective effect of EA against HFD-induced intestinal injury

To determine whether intestinal ILC2s are required for the beneficial effects of EA on HFD-induced intestinal injury, we selectively ablated intestinal ILC2s in obese mice and examined the resulting changes in type 2 cytokine production and intestinal histopathology. Following intestinal ILC2 ablation, the levels of type 2 cytokines IL-5 and IL-13 in the intestinal wall were significantly decreased (Proximal colon: **Figures 5A, B**; Distal colon: **Figures 5F,G**), and histopathological sections showed a significant reduction in goblet cell content, along with atrophy and a decline in the number of intestinal glands (Proximal colon: **Figures 5C–E**; Distal colon: **Figures 5H–J**). These pathological changes indicate that the protective effect of EA is largely abolished in the absence of intestinal ILC2s. Collectively, these findings demonstrate that intestinal ILC2s play an indispensable role in mediating EA-induced activation of type 2 immunity and the subsequent repair of high-fat diet-induced intestinal injury (**Figure 6**).

**Figure 5:**
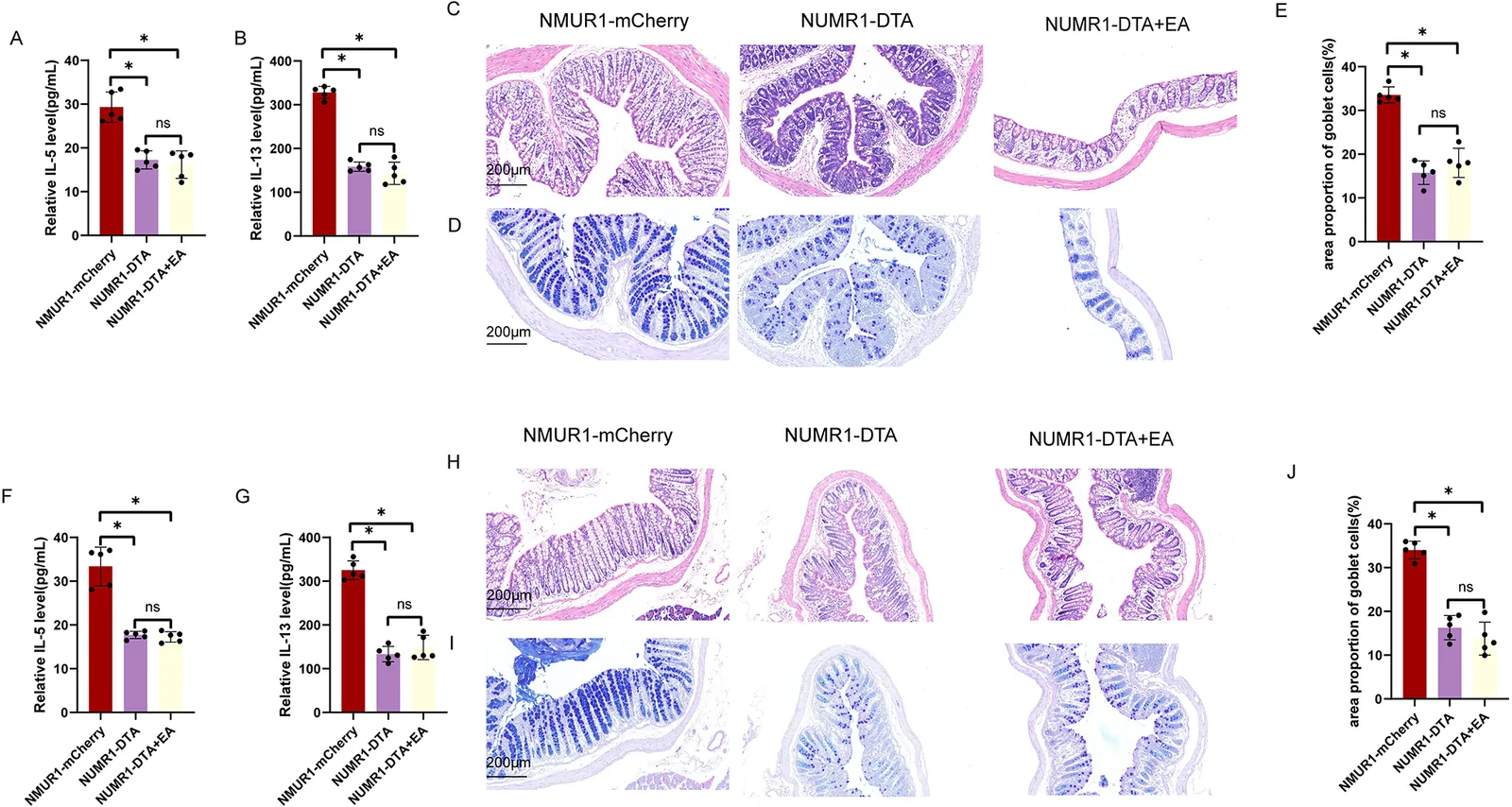
Ablation of intestinal ILC2s did not relieve intestinal injury caused by a high-fat diet. **(A)** Relative IL-5 expression in the proximal colon of NMUR1-mCherry, NMUR1-DTA, and NMUR1-DTA+EA groups. **(B)** Relative il-13 expression in the proximal colon of NMUR1-mCherry, NMUR1-DTA, and NMUR1-DTA+EA groups. **(C)** Representative H&E staining images of the proximal colon in NMUR1-mCherry, NMUR1-DTA, and NMUR1-DTA+EA groups. **(D, E)** Representative AB-PAS staining images and quantification of goblet cell ratio in the proximal colon of NMUR1-mCherry, NMUR1-DTA, and NMUR1-DTA+EA groups. **(F)** Relative IL-5 expression in the distal colon of NMUR1-mCherry, NMUR1-DTA, and NMUR1-DTA+EA groups. **(G)** Relative il-13 expression in the distal colon of NMUR1-mCherry, NMUR1-DTA, and NMUR1-DTA+EA groups. **(H)** Representative H&E staining images of the distal colon in NMUR1-mCherry, NMUR1-DTA, and NMUR1-DTA+EA groups. **(I, J)** Representative AB-PAS staining images and quantification of goblet cell ratio in the distal colon of NMUR1-mCherry, NMUR1-DTA, and NMUR1-DTA+EA groups. All n=5. **(C, D,H, I)** Scale bar =200 µm. Error bars represent mean ± SEM. Data were analyzed by one-way ANOVA (Tukey’s HSD *post hoc* test). ns indicates no significant difference; *p < 0.05.

**Figure 6:**
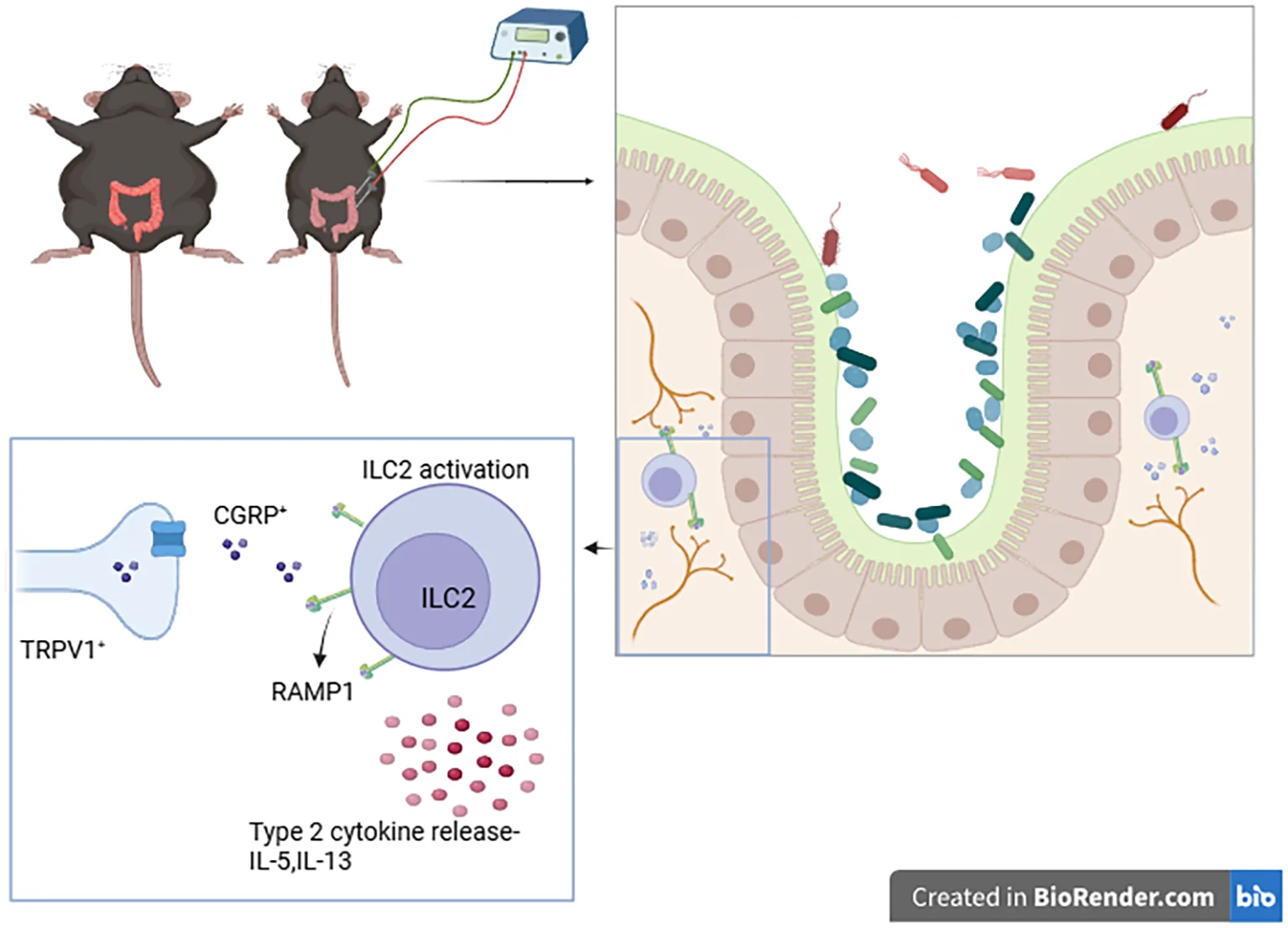
Mechanism schematic diagram.

## Discussion

Our findings demonstrate that electroacupuncture at ST25 effectively ameliorates obesity-induced intestinal injury. This protective effect is associated with the activation of the intestinal type 2 immune response via the TRPV1-CGRP-RAMP1 axis, which promotes the release of the type 2 cytokines IL-5 and IL-13.

High-fat diet-induced obesity increases intestinal barrier permeability, facilitating the translocation and leakage of pathogenic microorganisms and other harmful substances (18). ILC2s are core effector cells of intestinal innate immunity. Their membranes express various neuropeptide and neurotransmitter receptors, such as CRLR/RAMP1, NMUR1, and β2AR, enabling them to integrate epithelial and neuronal signals to maintain barrier tissue homeostasis. Activated ILC2s produce type 2 cytokines, interacting with other cells or directly promoting tissue damage repair (19). The regulation of ILC2s by TRPV1-expressing neurons exhibits tissue specificity, observed specifically in the colon but not in the ileum or cecum. Under steady-state conditions, persistent chemogenetic activation of TRPV1^+^ neurons significantly reduces the proportion of colonic ILC2s and suppresses the type 2 immune response (12). However, how TRPV1 regulates ILC2s and its role in intestinal homeostasis under HFD conditions remained unclear. Our study indicates that under obese conditions, CGRP derived from TRPV1-positive neurons in colonic tissue activates ILC2s via RAMP1. In the HFD model, the expression of ILC2-secreted cytokines IL-5 and IL-13 was downregulated, accompanied by reduced goblet cell numbers, leading to intestinal injury. Electroacupuncture increased the expression of intestinal sensory nerve fibers, ameliorated HFD-induced intestinal damage, activated the intestinal type 2 immune response, and promoted an increase in goblet cells. Ablation of TRPV1^+^ DRG neurons using RTX suppressed the intestinal type 2 immune response, exacerbated intestinal injury, significantly decreased goblet cell numbers, and inhibited the efficacy of EA. Administration of the RAMP1 antagonist BIBN4096 to obese mice significantly reduced ILC2 abundance, suppressed the colonic type 2 immune response, and worsened intestinal injury, thereby blocking the effect of EA. Research by XL also demonstrated that dietary succinate mediates goblet cell recovery by inducing type 2 immune sensing, improving HFD-induced small intestinal mucosal barrier injury and maintaining intestinal homeostasis (20). Beyond the gut, ILC2s are significantly reduced in adipose tissue during obesity (21), potentially due to chronic inflammation (e.g., macrophage and CD8^+^ T cell infiltration) inhibiting ILC2 survival or proliferation. ILC2s secrete IL-5 to maintain adipose tissue eosinophil survival and recruitment and secrete IL-13 to promote the accumulation of alternatively activated macrophages (AAMs). Eosinophils and AAMs can suppress adipose tissue chronic inflammation and enhance insulin sensitivity, thereby promoting fat metabolism and maintaining metabolic homeostasis (22). Furthermore, ILC2s can directly promote white adipose tissue browning by secreting methionine-enkephalin, preventing excessive fat accumulation (21).

As a key mediator of neuro-immune communication, CGRP not only relays peripheral sensory information to the central nervous system but also directly acts on immune cells, precisely regulating local immune responses. CGRP signaling can elicit both pro-inflammatory and anti-inflammatory immune effects, with this functional diversity determined by the cell type involved, target tissue characteristics, and the animal model context. Previous studies have established that CGRP can activate or suppress type 2 immunity in a dose- and context-dependent manner (23–25). Furthermore, different CGRP family members form distinct neuropeptide-receptor complexes (e.g., CALCRL-RAMP1 forms CGRPR, CALCRL-RAMP2 forms ADM1R, CALCRL-RAMP3 forms ADM2R), which can differentially modulate tissue immune responses (26). For instance, under specific stimuli, CGRP can inhibit ILC2 proliferation (23); whereas the CGRP-related peptide adrenomedullin (ADM) can directly enhance ILC2-mediated lung inflammation (27); and enteric neuron-derived adrenomedullin 2 (ADM2), acting via the ADM2 receptor pathway, promotes the colonic ILC2 response and mediates tissue protection in a mouse colitis model and in patients with inflammatory bowel disease (IBD) (28). In our study, CGRP derived from DRG TRPV1-expressing neurons promoted the colonic type 2 immune response in obese mice, providing new clues to the regulatory mechanisms of intestinal immune homeostasis in obesity. Consistent with this, findings from Jazib Uddin et al. showed that pathway analysis based on differentially expressed genes between control and CGRP-activated ILC2s revealed enrichment of genes related to tissue repair pathways, including upregulation of genes associated with the damage response and epidermal growth factor receptor signaling (28). However, in studies of pulmonary immunity, the neuropeptide subtype CGRPβ, encoded in vagal ganglia, inhibits ILC2 activation and allergic lung inflammation, promoting pulmonary immune homeostasis (8). Under homeostatic conditions, α-CGRP produced by choline acetyltransferase-positive (CHAT^+^) enteric neurons is a crucial neuropeptide for maintaining the homeostasis of small intestinal KLRG1^+^ ILC2s. Upon induction of allergic inflammation by ovalbumin (OVA) or direct activation of the inflammatory pathway using IL-25, the small intestine rapidly initiates a type 2 inflammatory response, characterized by a sharp increase in KLRG1^+^ ILC2 numbers. Administration of α-CGRP significantly suppressed KLRG1^+^ ILC2 abundance. Additionally, ILC2s themselves secrete CGRP in an autocrine manner upon IL-25 stimulation (25). Nagashima et al. (23) demonstrated that CGRP inhibited ILC2 proliferation and IL-13 production while promoting IL-5 expression in the lung during helminth infection and IL-33-induced airway inflammation, thereby constraining the overall type 2 immune response. In contrast, our study shows that CGRP derived from TRPV1+ DRG neurons enhances colonic ILC2 accumulation and type 2 cytokine production (IL-5 and IL-13), ultimately ameliorating obesity-induced intestinal barrier injury. This apparent discrepancy may be related to differences in tissue microenvironment and disease state. First, the lung is a barrier organ constantly exposed to airborne allergens and pathogens, where excessive type 2 inflammation would compromise gas exchange; therefore, CGRP serves to limit ILC2-driven pathological responses in this context. In contrast, the colon under obese conditions is characterized by metabolic inflammation, disrupted tight junctions, and microbial translocation. Under these circumstances, a moderate enhancement of type 2 immunity may be required to support goblet cell differentiation, mucus production, and epithelial repair. Second, the source and concentration of CGRP differ substantially. Nagashima et al. demonstrated that ILC2s themselves can produce CGRP (autocrine) and used a relatively high concentration of exogenous CGRP (10 ng/mL *in vitro* and 1 µg/mouse *in vivo*). In our study, CGRP is released from TRPV1+ sensory nerve terminals innervating the colonic wall, likely acting at lower, spatially confined concentrations, which may lead to distinct signaling outcomes. Third, the inflammatory context diverges: helminth infection triggers a strong acute type 2 response that requires a braking mechanism, whereas obesity-induced intestinal injury involves chronic low-grade inflammation with a predominant repair deficit. Thus, CGRP may act as a context-dependent rheostat – suppressing excessive inflammation in one setting while promoting tissue-protective immunity in another. Fourth, receptor availability and downstream signaling may be modulated by the local milieu. Nagashima et al. showed that CGRP acts through the canonical CLR-RAMP1 receptor complex to increase cAMP, thereby inhibiting NMU- and IL-33-driven proliferation and IL-13 expression. In our obese colon model, we found that RAMP1 signaling is involved; however, whether additional receptor components (such as RAMP2 or RAMP3) are engaged or alter the cellular response remains to be determined.

TRPV1^+^ neurons play a vital role in intestinal immune homeostasis by sensing signals from the intestinal microenvironment, regulating immune cell activation and cytokine secretion, suppressing excessive inflammation, and enhancing anti-infective capacity. Silencing TRPV1 globally or specifically in the colon using chemogenetic or pharmacological approaches leads to increased inflammatory cell infiltration, significant goblet cell loss, and crypt structure destruction (9). Conversely, activating TRPV1 via chemogenetics, natural ligands (e.g., capsaicin), or physiological stimuli (e.g., commensals/pathogens) confers intestinal protection (9, 10, 29). Nociceptor TRPV1 can directly regulate intestinal immune cells. Interestingly, TRPV1^+^ sensory neurons in the DRG mediate a reduction in intestinal RORγ^+^ Treg cell numbers via the CGRP-RAMP1 signaling pathway; activating these neurons exacerbates colitis, barrier damage, and pathogen susceptibility during the recovery phase (but not the initiation phase) in a mouse colitis model (12). This discrepancy might stem from differences in activation methods. Furthermore, nociceptor TRPV1 mediates mucosal protection via communication with intestinal epithelial cells. TRPV1^+^ nociceptors in the DRG can release CGRP to regulate the density of M cells in ileal Peyer’s patches, inhibiting bacterial infection, and upregulate the levels of segmented filamentous bacteria, thereby providing further protection against Salmonella typhimurium infection (29). Nav1.8^+^ nociceptors drive goblet cells to release mucus via CGRP, reinforcing the mucus layer defense and enhancing the protective function of the intestinal barrier (10). TRPV1 nociceptors can also modulate gut microbiota composition via substance P (SP), promoting the proliferation of beneficial bacteria (e.g., Clostridium spp.) and suppressing the overgrowth of pathogens, thereby maintaining intestinal homeostasiss (9). In summary, our study demonstrates that electroacupuncture ameliorates high-fat diet-induced intestinal barrier injury by activating the type 2 immune response in the gut through TRPV1-CGRP-RAMP1 signaling, thereby promoting goblet cell function in obese mice.

Acupuncture may ameliorate intestinal inflammation through multiple mechanisms. First, it restores tight junction proteins to strengthen the intestinal physical barrie (30), thereby reducing pathogen translocation. Second, acupuncture modulates the composition of the gut microbiota (31–34), promoting the proliferation of beneficial bacteria such as Lactobacillus and the production of their metabolite, short-chain fatty acids, which contributes to intestinal homeostasis. In addition, acupuncture activates a vagus nerve-dependent but spleen-independent neural circuit (35), protecting against intestinal inflammation and dysmotility. These mechanisms collectively represent a multi-targeted therapeutic approach for managing intestinal inflammation.

The study by Liu et al. (36) revealed the neuroanatomical basis through which electroacupuncture at limb acupoints (e.g., ST36 at the hindlimb) exerts systemic anti-inflammatory effects via the vagal-adrenal axis. This pathway relies on the specific innervation of deep fascia (e.g., periosteum and intramuscular fascia) by PROKR2^+^ somatosensory neurons and can be activated by low-intensity electroacupuncture (0.5 mA). Notably, 63.9% of PROKR2^+^ DRG neurons co-express CGRP, and approximately 50% of them also co-express NEFH; these CGRP^+^PROKR2^+^ neurons are key populations innervating deep fascia. In sharp contrast, the present study demonstrates that electroacupuncture at the abdominal ST25 acupoint activates TRPV1^+^CGRP^+^ sensory neurons to regulate local type 2 immune responses in the intestine, thereby repairing obesity-induced colonic barrier damage. Anatomical evidence shows that PROKR2^+^ fibers are barely distributed in the peritoneum of the abdominal ST25 region, forming only circumferential endings around hair follicles, which is consistent with our observation that the effects at ST25 depend on TRPV1 rather than PROKR2. In summary, the effects of electroacupuncture at different acupoints are dictated by their unique innervation patterns: limb acupoints produce systemic anti-inflammatory effects via the PROKR2^+^ deep fascia-vagal pathway, whereas abdominal acupoints regulate intestinal local homeostasis through the TRPV1^+^ visceral afferent-local ILC2 pathway.

## Limitations of the study

We acknowledge several limitations in the current study. The experimental design was based solely on a single high-fat diet-induced obesity mouse model. While these numbers were sufficient to detect primary mechanistic effects, they preclude broad generalization. Although, clinical studies have shown that acupuncture is effective treatment for obesity and inflammatory bowel disease (37–39). A key limitation is the absence of validation human samples in obesity with intestinal inflammation. Therefore, while our data provide novel insights into intestinal dysfunction in the context of diet-induced obesity, any implications for human therapy remain preliminary and hypothetical. Furthermore, since RAMP1 is widely expressed in vascular endothelial cells, neurons, and epithelial cells, to elucidate the cell-specific mechanism, future studies should employ ILC2-specific RAMP1 conditional knockout mice to further clarify the mechanism of action. This study has several methodological limitations. Because the normal diet group did not receive electroacupuncture or pharmacological intervention, the study lacks a “normal diet + intervention” control, making it impossible to determine whether electroacupuncture or pharmacological treatment can restore the relevant parameters to normal levels or to exclude the potential influence of dietary conditions themselves on the observed changes. Future studies should include a normal diet intervention control group and adopt more rigorous randomization and blinding designs.

## Data Availability

The original contributions presented in the study are included in the article/**Supplementary Material**. Further inquiries can be directed to the corresponding authors.

## Supplementary material

Supplementary Figure 1 Electroacupuncture ameliorates lipid metabolism disorders in obese mice. **(A)** Wet weight of inguinal white adipose tissue (unilateral) in ND, HFD, and EA groups. **(B)** Wet weight of epididymal white adipose tissue (unilateral) in ND, HFD, and EA groups. **(C–H)** Serum levels of free fatty acids (NEFA), triglycerides (TG), total cholesterol (T-CHO), low-density lipoprotein cholesterol (LDL-C), high-density lipoprotein cholesterol (HDL-C), and glycerol in ND, HFD, and EA groups. All n=5. Error bars represent mean ± SEM. Statistical significance was determined by one-way ANOVA (Tukey’s HSD *post hoc* test). *p < 0.05.

Supplementary Figure 2 Immunofluorescence detection of RAMP1, TRPV1, and CGRP in the mouse colon. **(A)** Representative immunofluorescence images of RAMP1 (purple) in the proximal colon of HFD + Vehicle and BIBN4096 groups. **(B)** Representative immunofluorescence images of RAMP1 (purple) in the distal colon of HFD + Vehicle and BIBN4096 groups. **(C)** Representative immunofluorescence images of TRPV1 (green) and CGRP (red) in the proximal colon of Scrambled shRNA, TRPV1 shRNA groups. **(D)** Representative immunofluorescence images of TRPV1 (green) and CGRP (red) in the distal colon of Scrambled shRNA, TRPV1 shRNA groups.
