# Supplementary material for: Neuroimmune activation of the TRPV1–RAMP1 axis drives type 2 immunity to repair intestinal barrier and alleviate metabolic disease
Source: Front Immunol. 2026 Oct 7;17:1965520. doi: 10.3389/fimmu.2026.1965520 (PMC13683677; doi:10.3389/fimmu.2026.1965520)

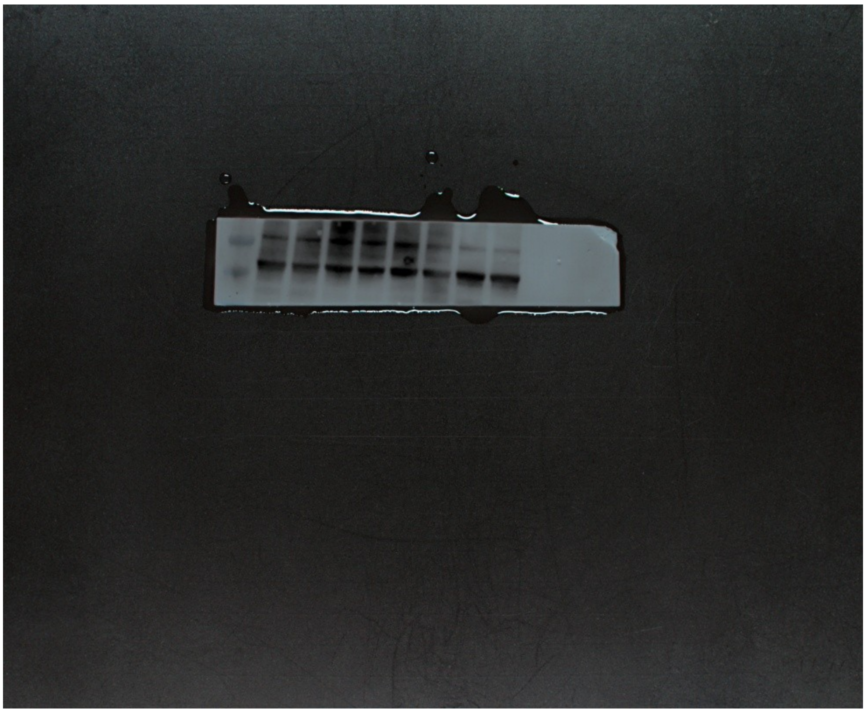

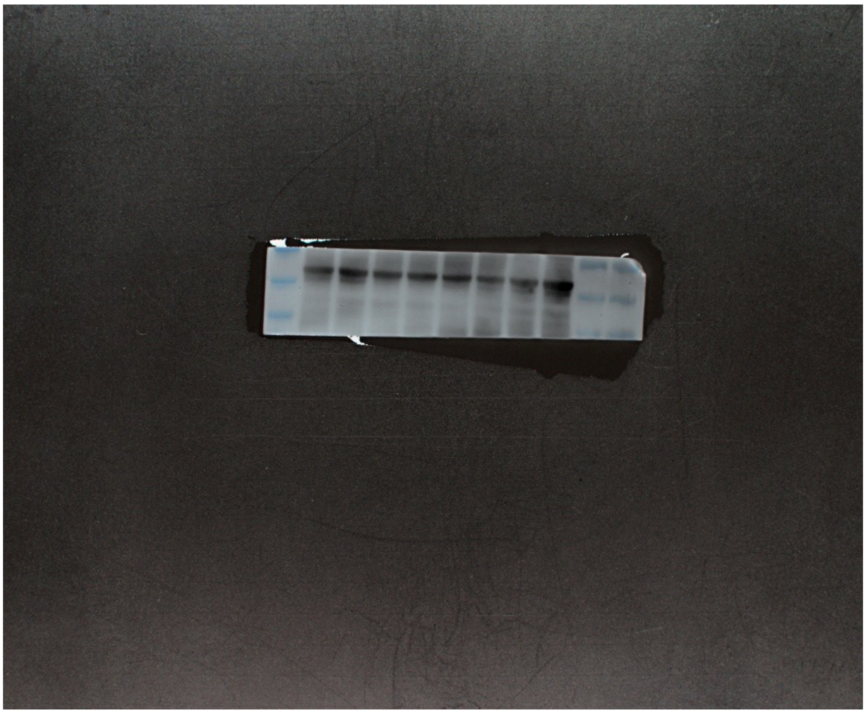

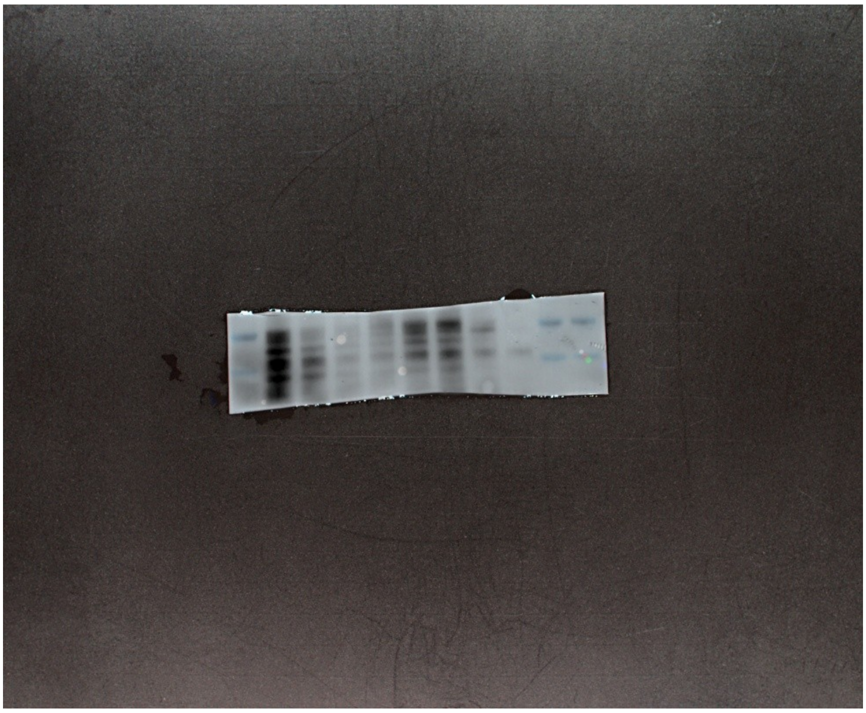

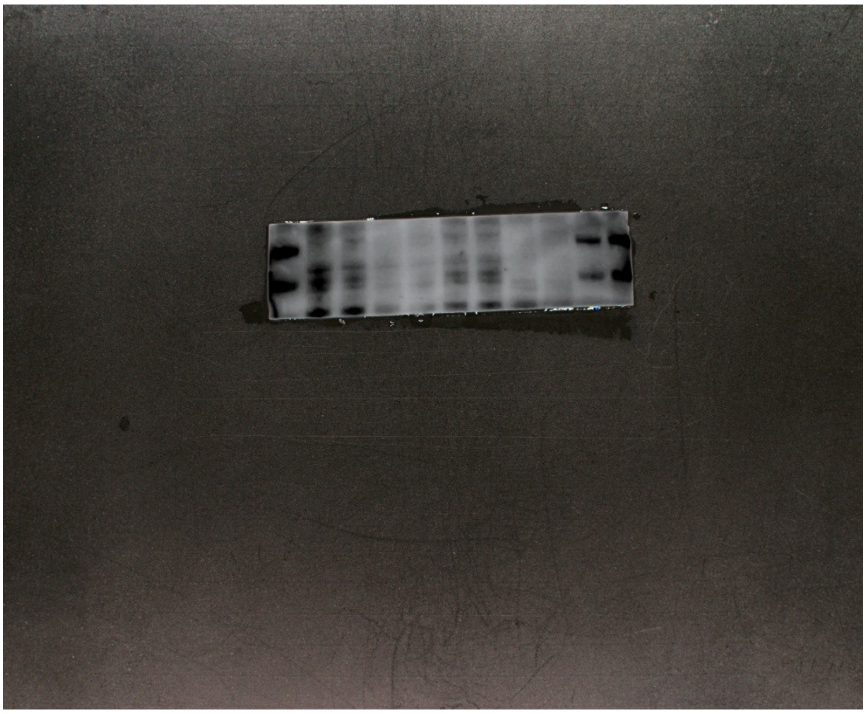

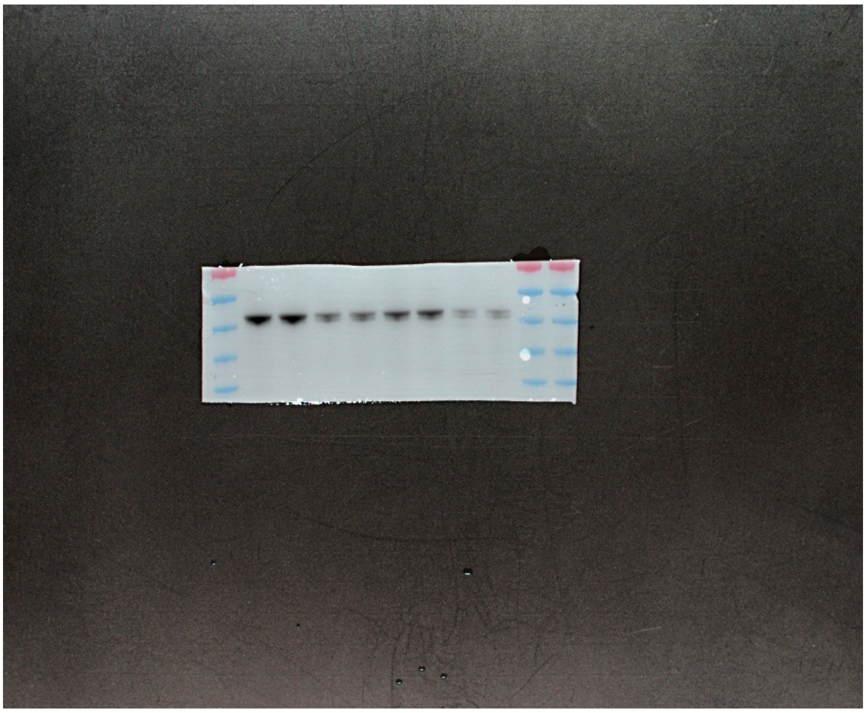

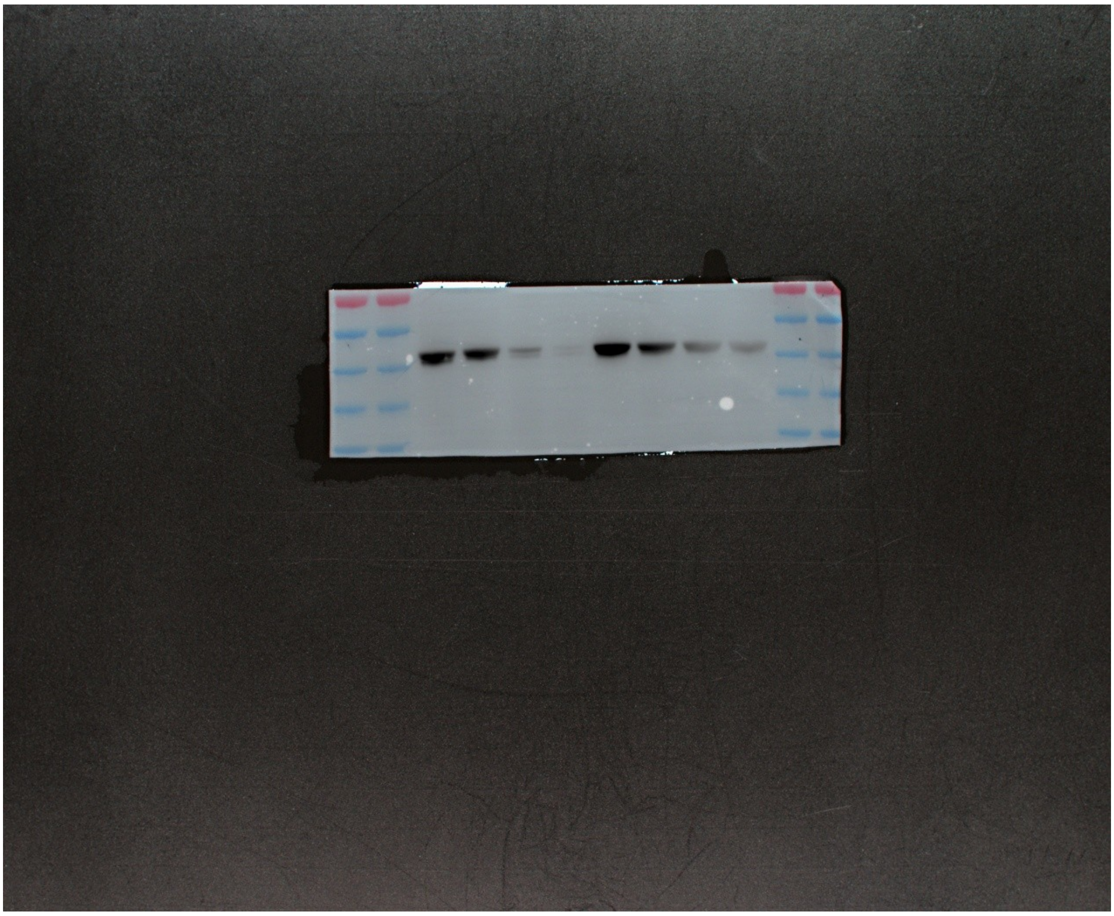

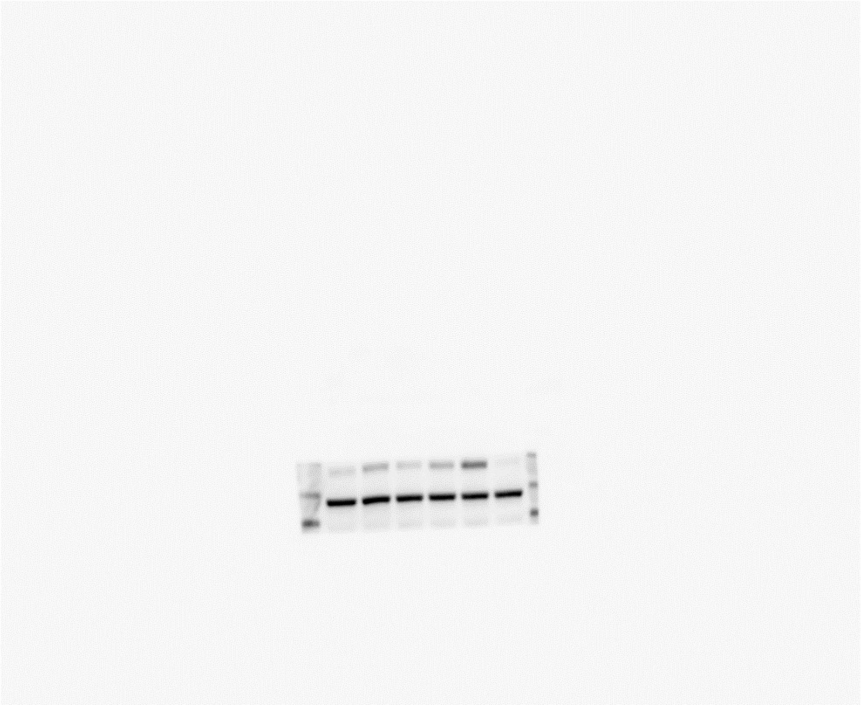

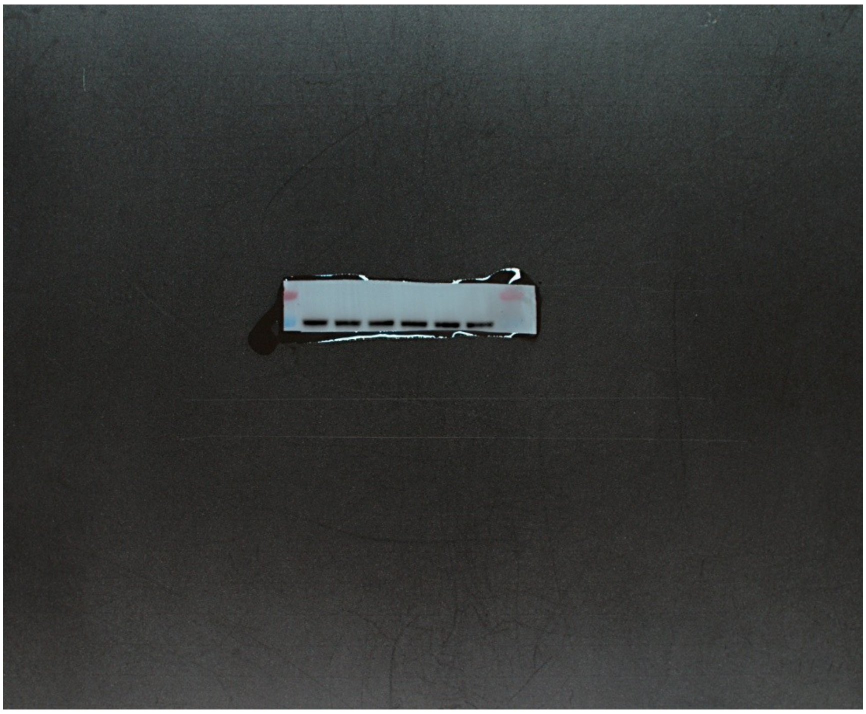

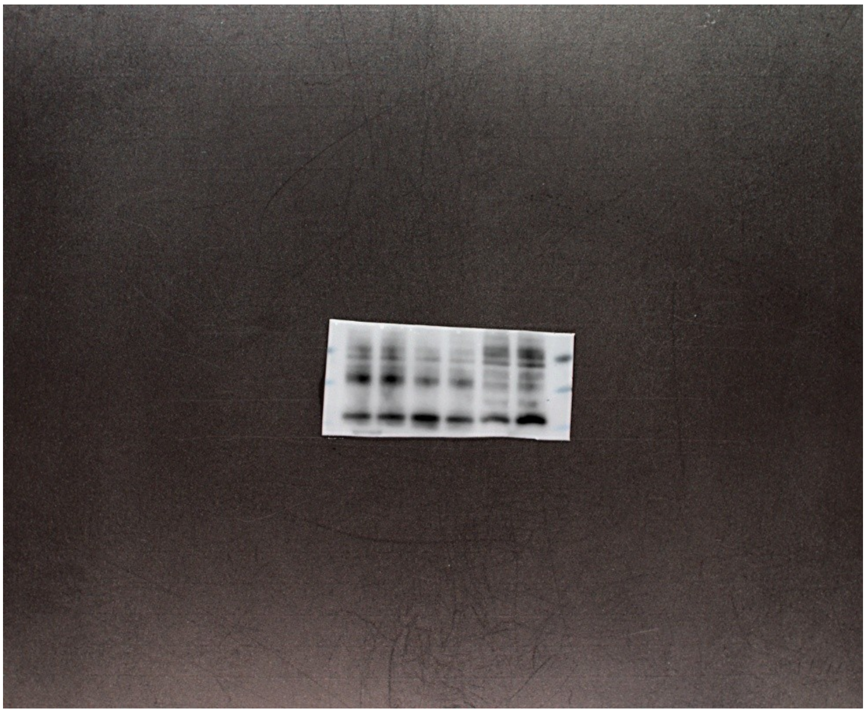

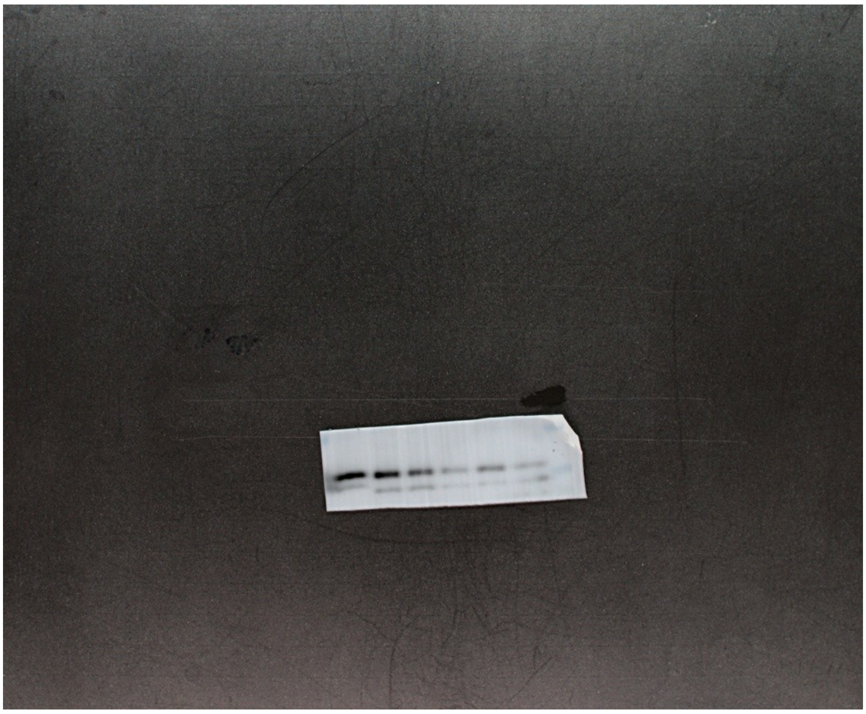

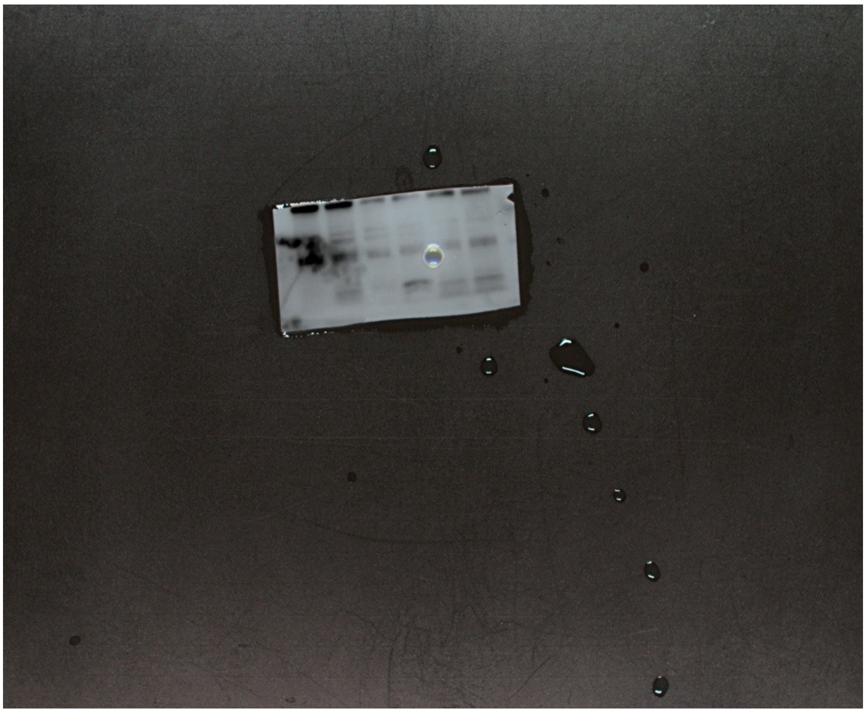

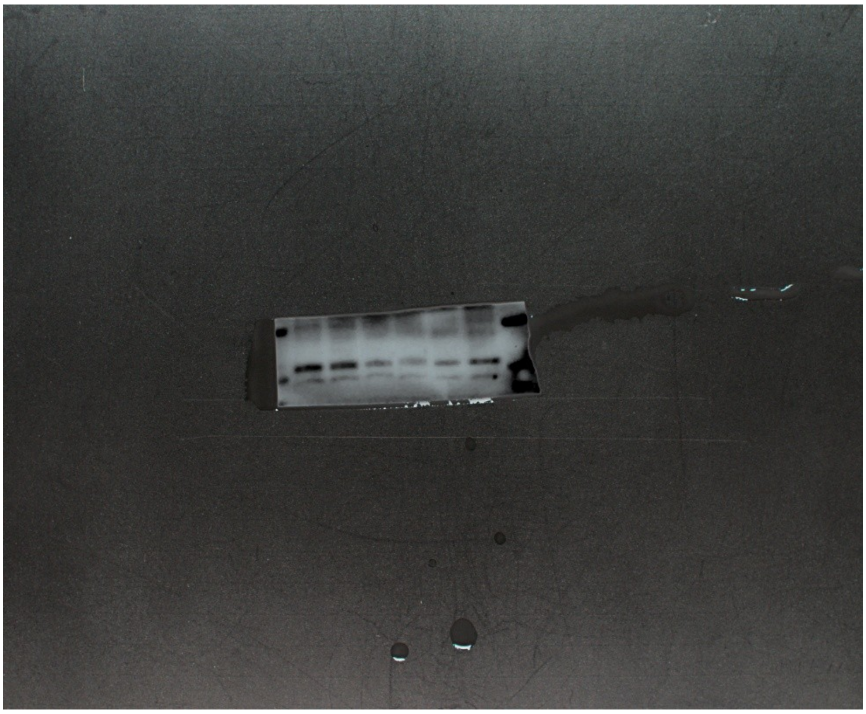

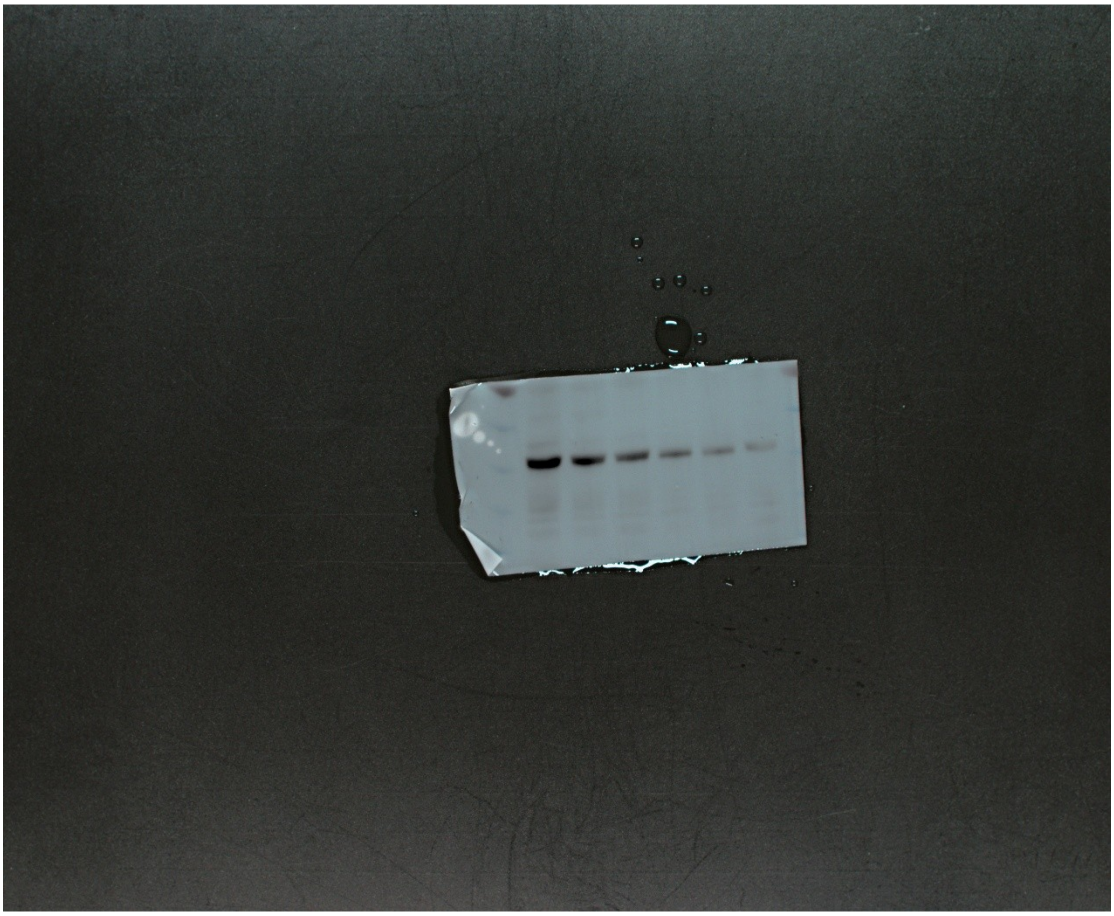

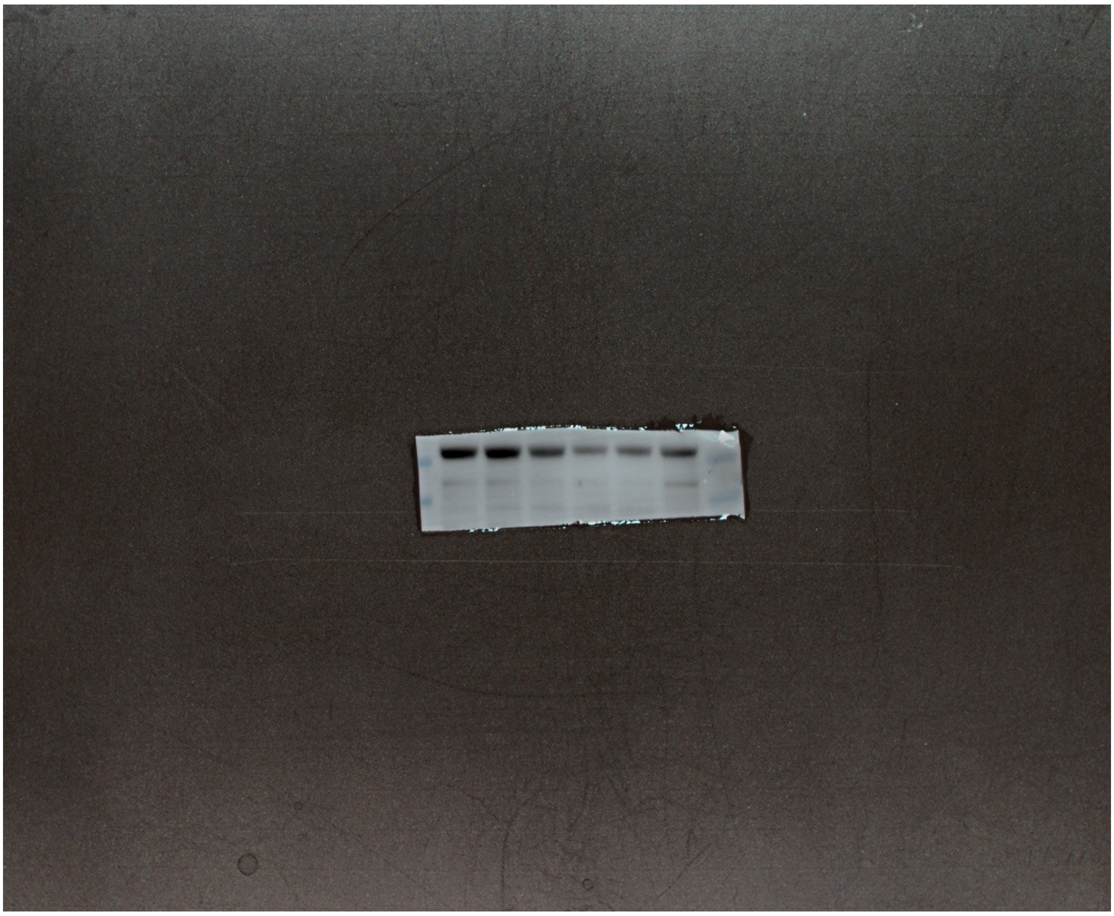

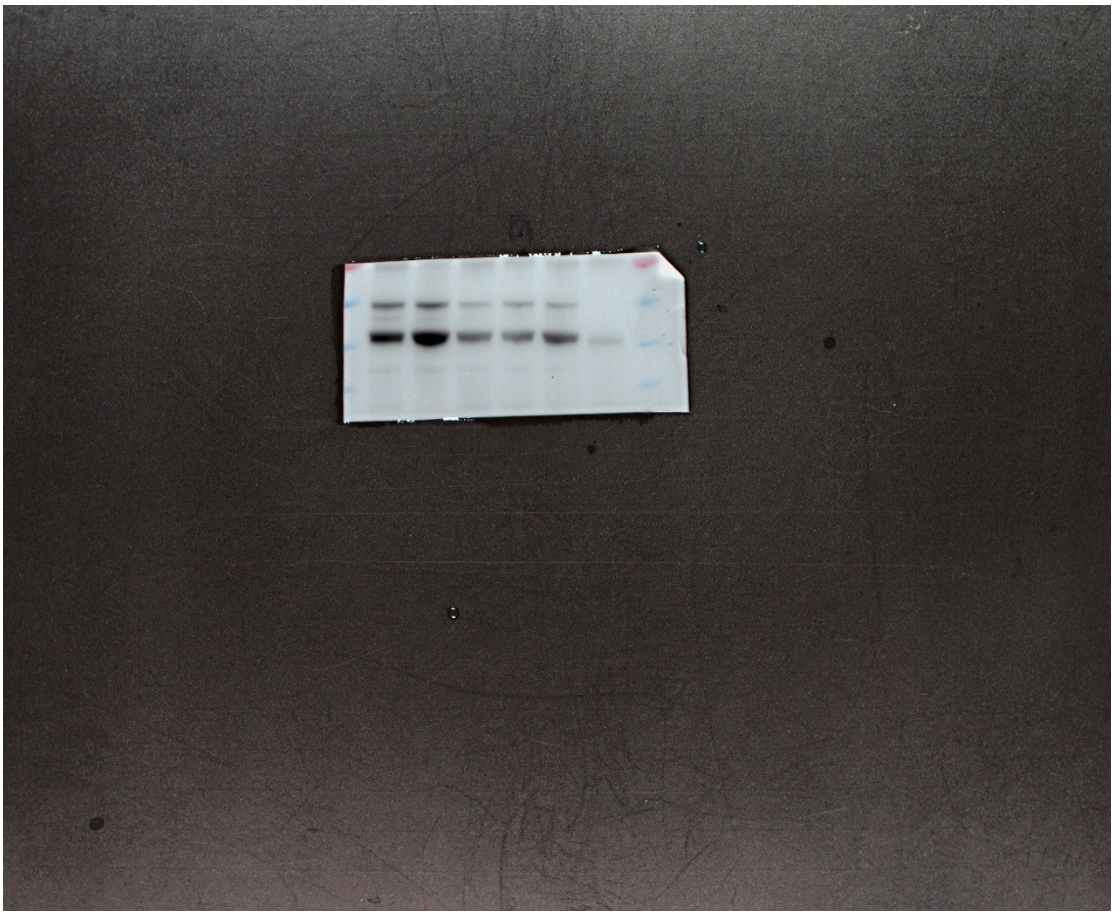

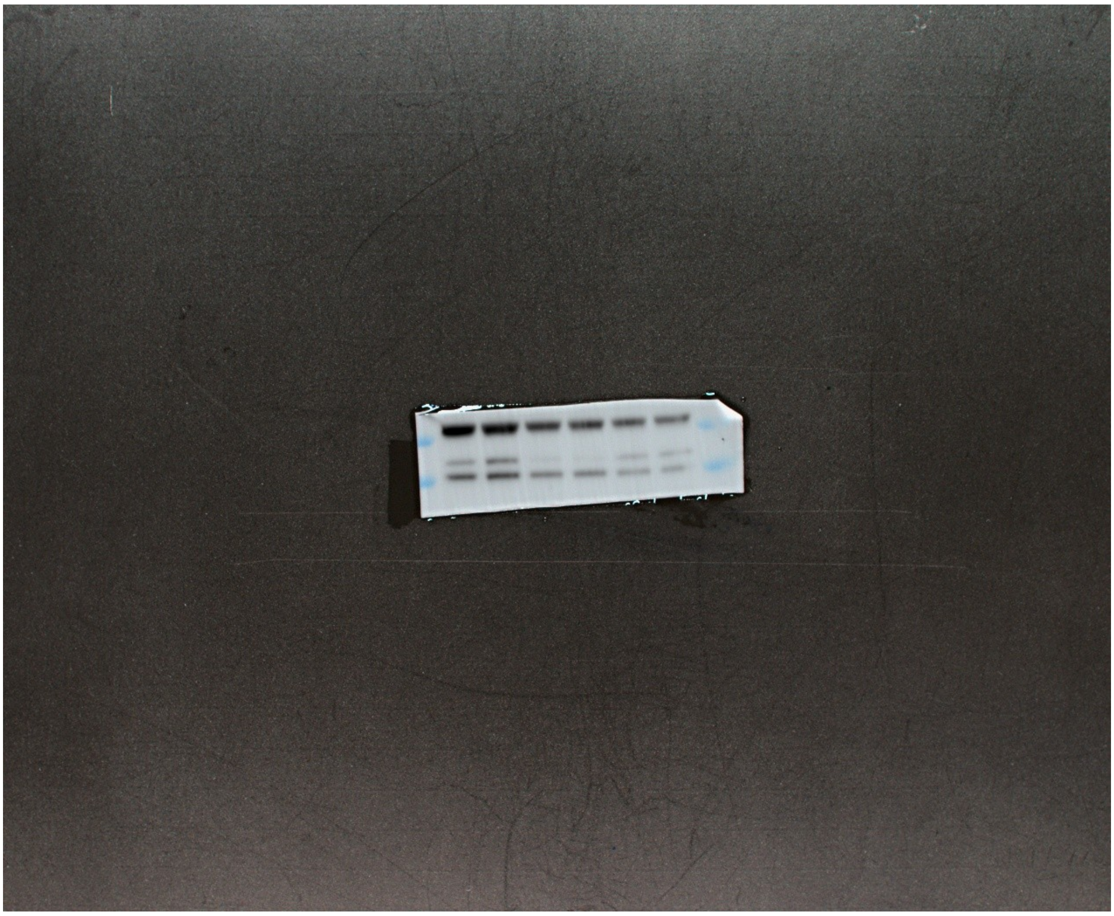

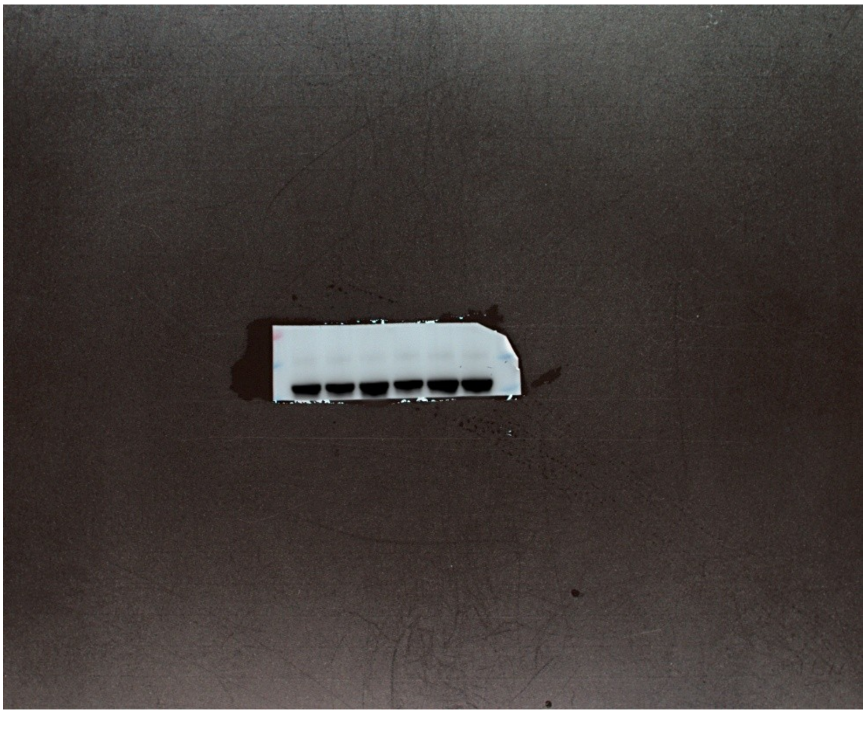

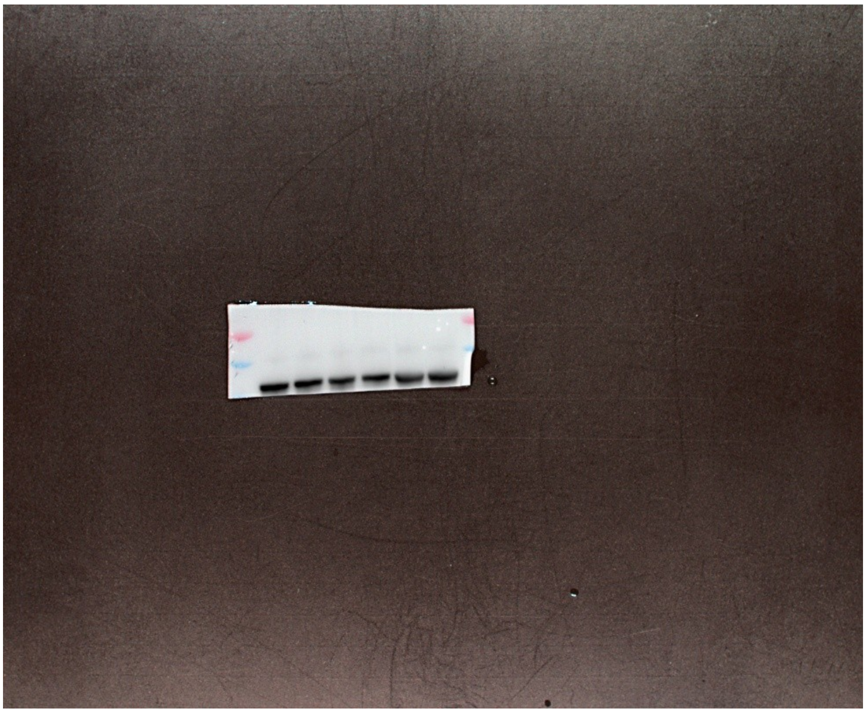

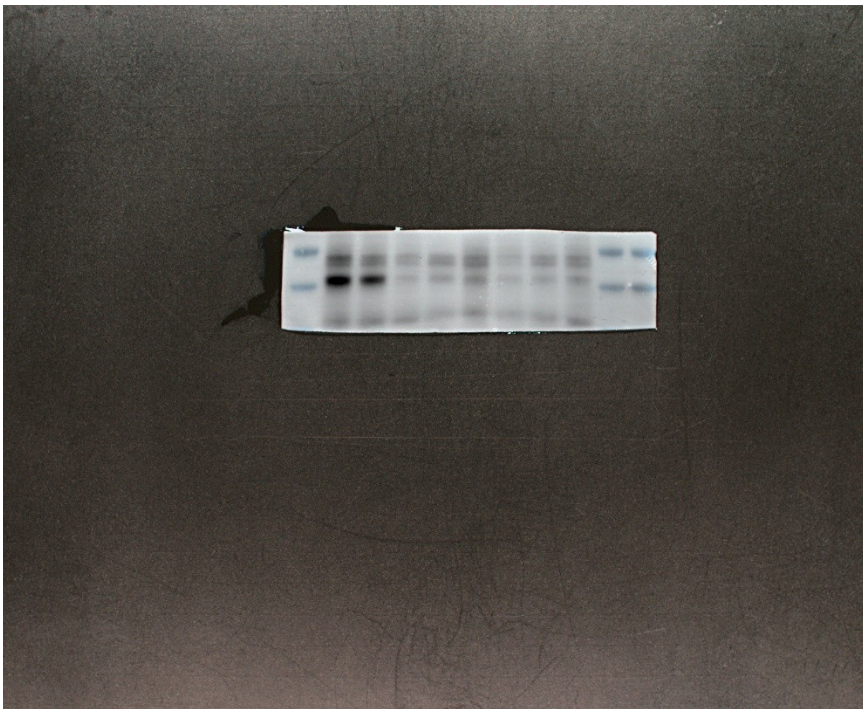

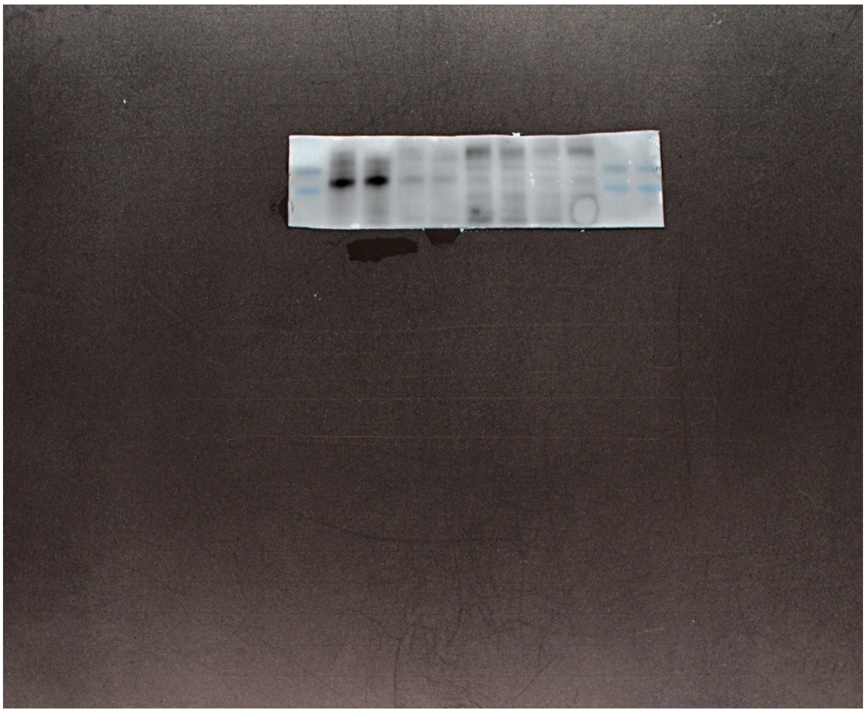

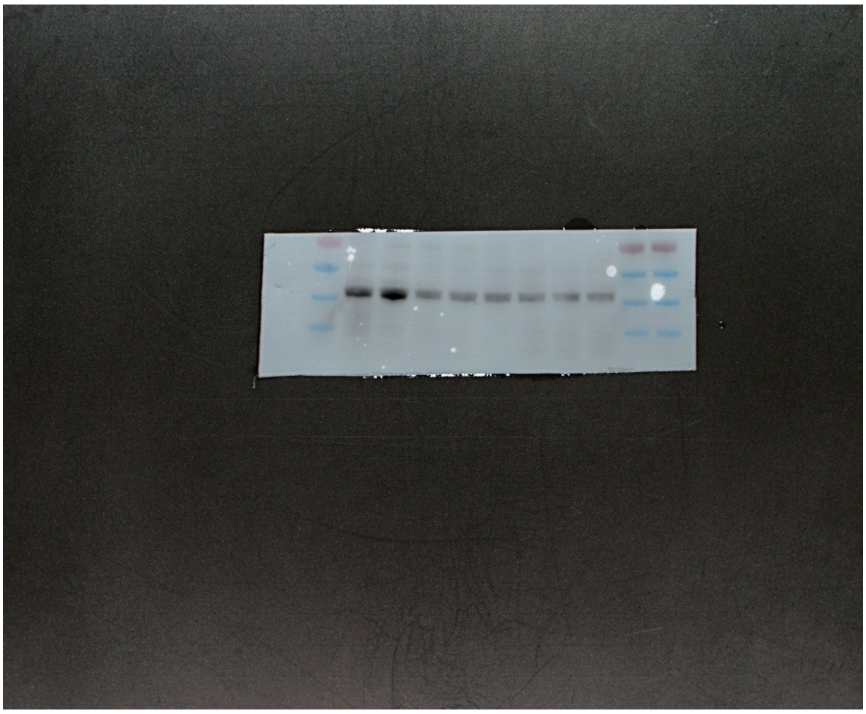

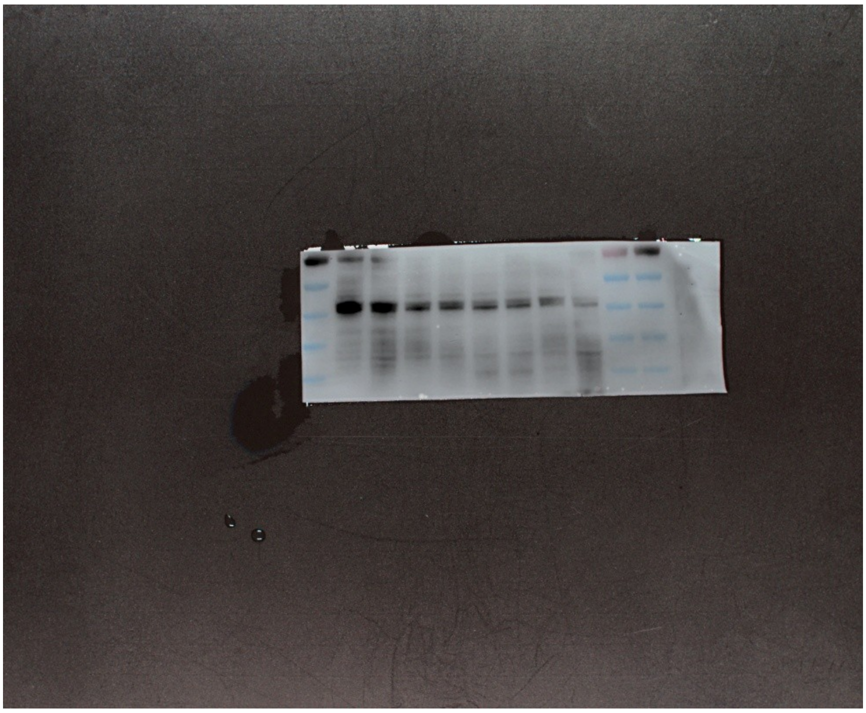

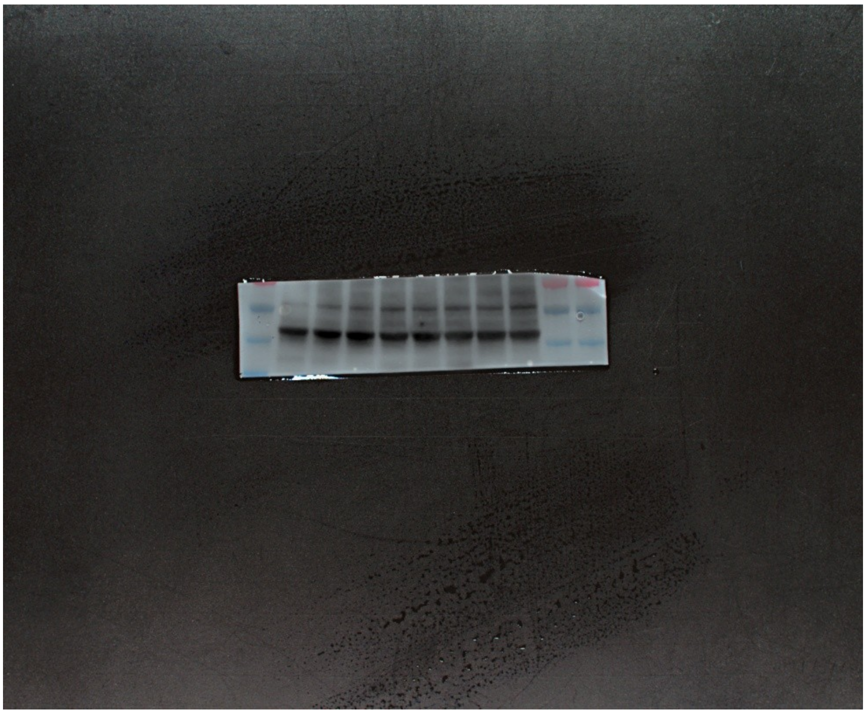

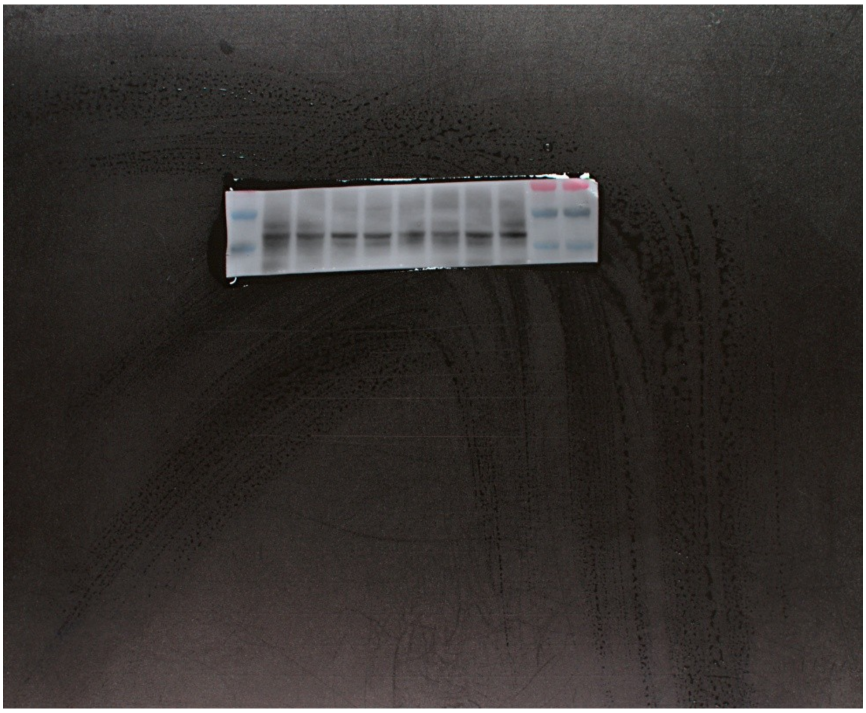

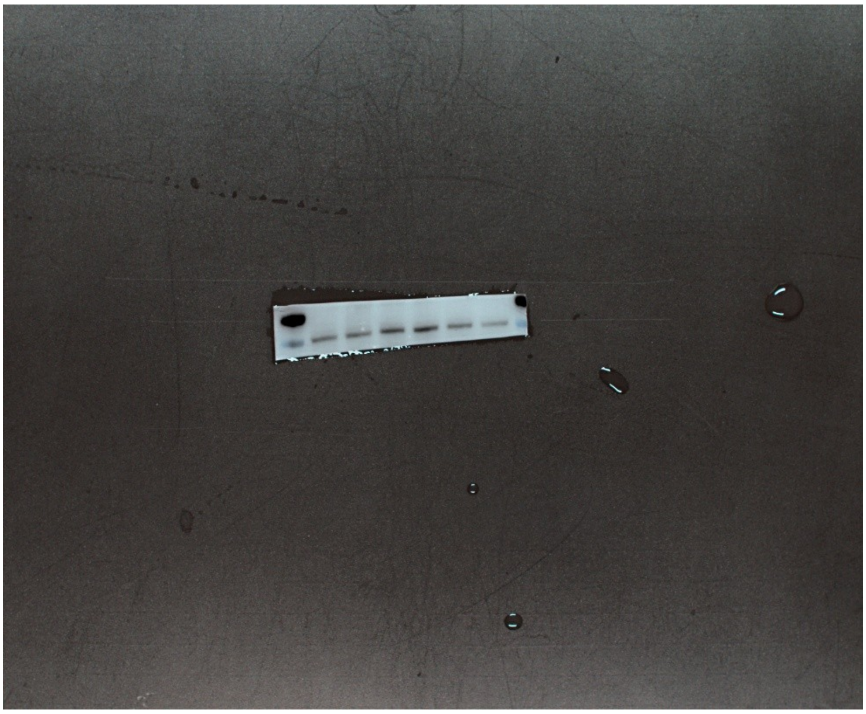

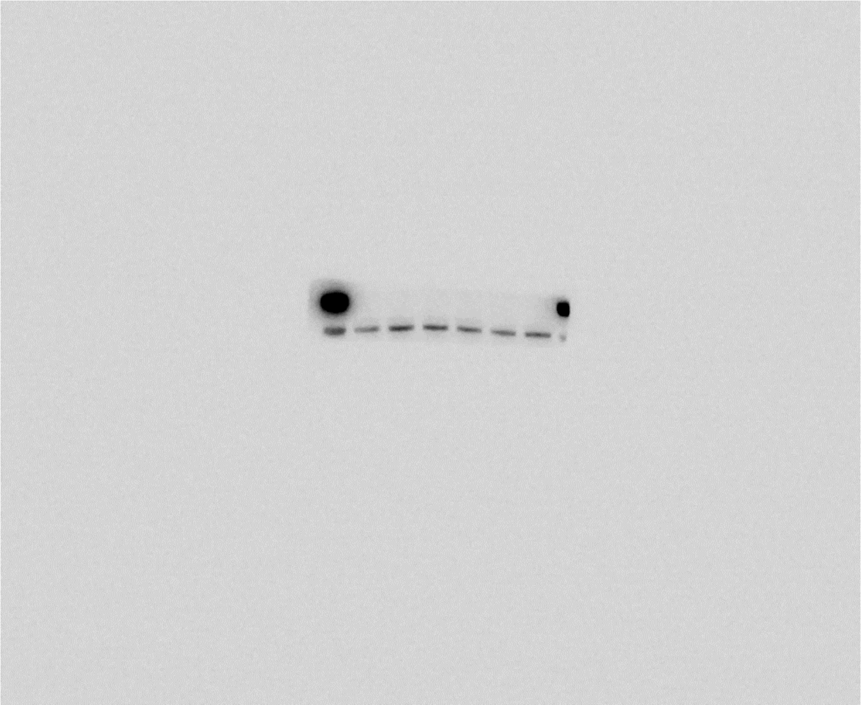

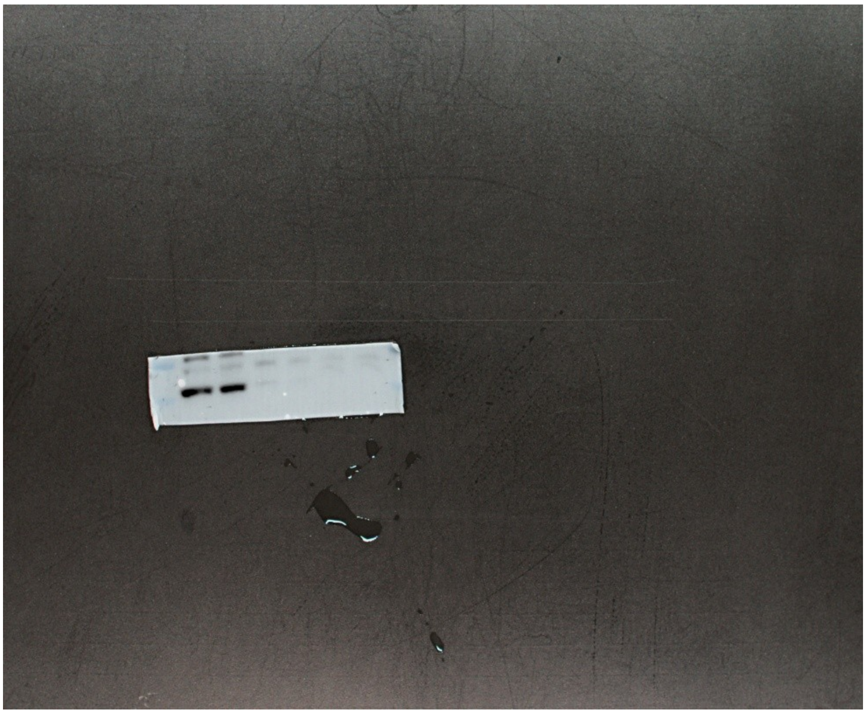

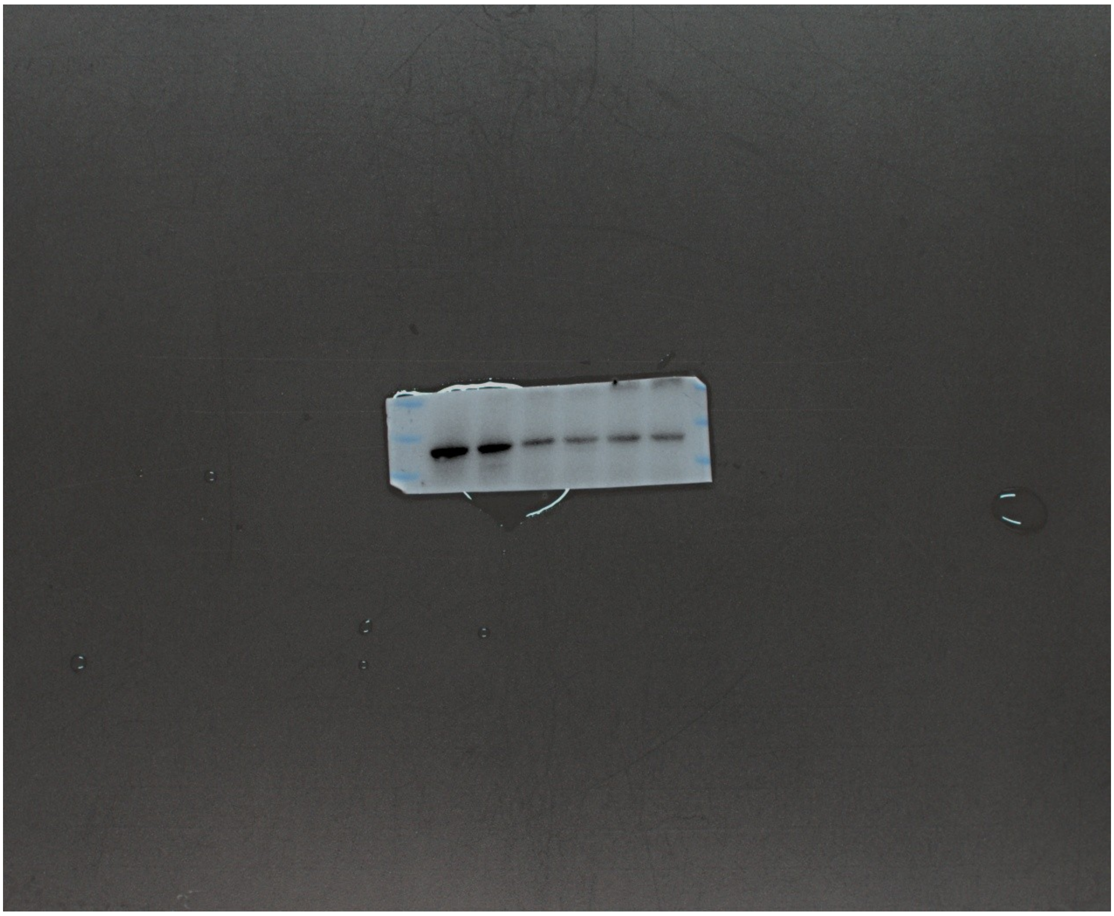

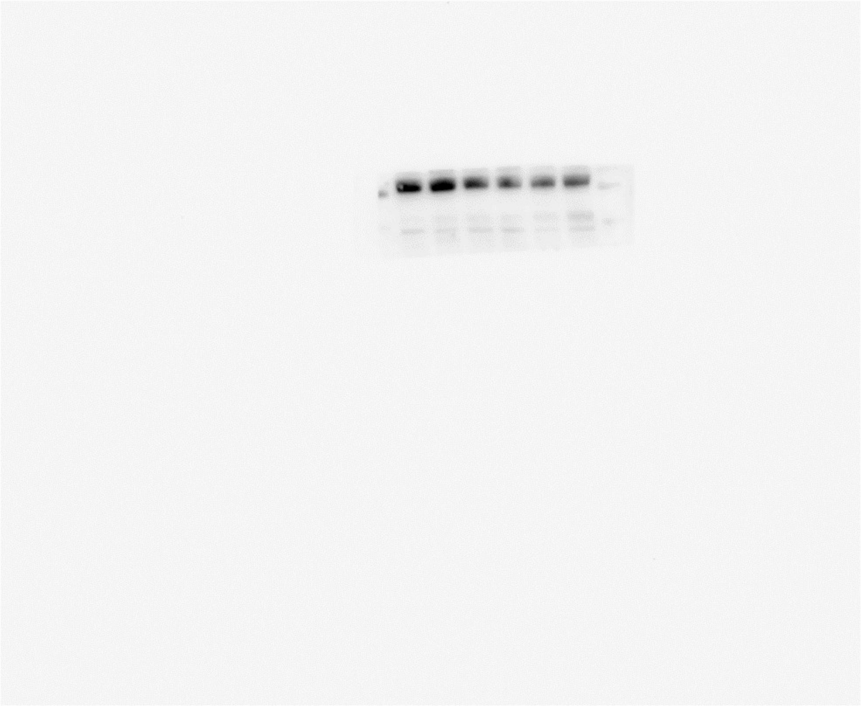

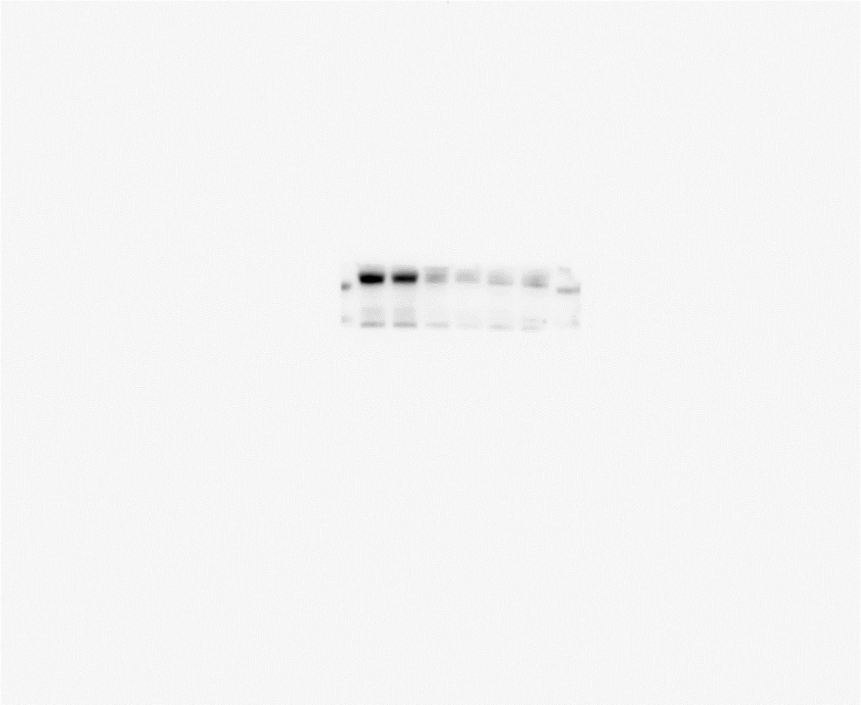

Supplement: Supplementary file 1 [file DataSheet1.docx]
